# Antibiotic-Free Gene Vectors: A 25-Year Journey to Clinical Trials

**DOI:** 10.3390/genes15030261

**Published:** 2024-02-20

**Authors:** Corinne Marie, Daniel Scherman

**Affiliations:** 1 Université Paris Cité, CNRS, Inserm, UTCBS, 75006 Paris, France; daniel.scherman@parisdescartes.fr; 2 Chimie ParisTech, Université PSL, 75005 Paris, France; 3 Fondation Maladies Rares, 75014 Paris, France

**Keywords:** antibiotic-free gene vectors, plasmids, transposons, non-viral gene therapy, CAR-T cells, CAR-NK cells, clinical trials

## Abstract

Until very recently, the major use, for gene therapy, specifically of linear or circular DNA, such as plasmids, was as ancillary products for viral vectors’ production or as a genetic template for mRNA production. Thanks to targeted and more efficient physical or chemical delivery techniques and to the refinement of their structure, non-viral plasmid DNA are now under intensive consideration as pharmaceutical drugs. Plasmids traditionally carry an antibiotic resistance gene for providing the selection pressure necessary for maintenance in a bacterial host. Nearly a dozen different antibiotic-free gene vectors have now been developed and are currently assessed in preclinical assays and phase I/II clinical trials. Their reduced size leads to increased transfection efficiency and prolonged transgene expression. In addition, associating non-viral gene vectors and DNA transposons, which mediate transgene integration into the host genome, circumvents plasmid dilution in dividing eukaryotic cells which generate a loss of the therapeutic gene. Combining these novel molecular tools allowed a significantly higher yield of genetically engineered T and Natural Killer cells for adoptive immunotherapies due to a reduced cytotoxicity and increased transposition rate. This review describes the main progresses accomplished for safer, more efficient and cost-effective gene and cell therapies using non-viral approaches and antibiotic-free gene vectors.

## 1. Introduction

Undoubtedly, the entire gene and cell therapy area has greatly benefited from the major breakthroughs that emerged over the last two decades. The completion of the first human and mammalian genome sequences [1,2], coupled with both omics data and the first draft of the human pangenome reference, created to capture the spectrum of genomic variation across the human population [3,4], has provided highly fundamental and valuable information for the characterization of genetic diseases. In addition, the emergence and refinement of novel technologies used for genome editing or gene transfer has opened new avenues to propose alternative treatments to genetic diseases or partly curable diseases such as cancer. In parallel, the massive effort allocated to improve the therapeutic gene and expression vectors delivering foreign DNA into mammalian cells has resulted in the sprouting of the number of preclinical assays and clinical trials. In 2023, 3674 clinical trials were recorded worldwide in the Gene Therapy Clinical trials database (https://a873679.fmphost.com/fmi/webd/GTCT, accessed on 5 April 2023). This concerns all phases of clinical development, with the following proportion: Phase I accounts for the majority with 56.4% of the studies, followed with phase I/II (23.1%), phase II (14.9%), phase II/III (0.9%), phase III (4.2%) and Phase IV (0.1%).

Nucleic acid entry into eukaryotic cells requires a delivery vector that can be broadly designated as either non-viral or viral. Viral approaches exploit the transducing capacity of viruses depleted from their pathogenic and irrelevant traits. Crucial genetic viral information required for envelope synthesis, when applicable, nucleic acid replication and packaging are split over several genetic determinants, such as plasmids, to co-transfect a “producing cell line” which generates viral particles carrying the therapeutic genetic cassette, but which are devoid of infective and replication capacity. The most common viral vectors used for gene therapies are derived from adeno-, retro-, lenti-, and adeno-associated viruses (AAV). Each of these viral vector families displays specific properties, which are mostly related to their packaging DNA capability, the transgene episomal status or integrative nature into the host genome necessary to maintain transgene expression in dividing cells. Thanks to the extensive basic research dedicated to their optimization, viral vectors for the delivery of therapeutic genes held their promises which resulted in several commercialized gene therapy products [5]. However, the use of viral vectors for gene and cell therapy application may become limited due to their lengthy and costly production. This is especially the case for treating diseases affecting a large number of patients, or when a large mass of body tissue needs to be transduced, such as for spinal muscular atrophy or Duchenne myopathy, where usually more than 10^14^ viral genomes per kilogram of body weight are administered to patients [6].

Compared to viral gene vectors, non-viral gene vectors display attractive features such as an easier, faster and cheaper production, no transgene size limit, and an almost complete absence of immunogenicity. Nevertheless, despite these positive assets, non-viral gene vectors have, until recently, been hampered by various limitations such as (i) a low transfection efficiency of some specific cell types and primary cells, (ii) a lack of tissue or organ specificity, (iii) a decrease in transgene expression due to epigenetic silencing, mainly in hepatocytes, and (iv) a lack of vector maintenance in transfected dividing cells.

This review focuses on the major non-viral achievements accomplished to tackle some of the aforementioned limiting factors, including (i) the optimization of chemical and physical delivery methods for an increased transfection efficiency and specificity, (ii) the rationale for the development of a smaller and antibiotic-free gene vector, and (iii) the development of alternative antibiotic-free gene vectors, including Minicircles, MIDGE, DoggyBone^TM^, Gencircles^TM^, pORT, Nanoplasmids, pCOR and pFAR miniplasmids. In addition, we provide a table listing the ongoing and completed clinical trials conducted using the antibiotic-free gene vectors to either correct genetic defects in diseased cells or to introduce new genetic traits to elicit or boost protective immunity. The main characteristics of DNA transposons, such as *Sleeping Beauty (SB)*, *PiggyBac (PB)* and *TcBuster*, which mediate transgene integration into the host genome, are presented to highlight the mutual benefit of merging them with antibiotic-free gene vectors for the production of stable and genetically modified cells such as T or Natural Killer (NK) cells expressing a chimeric antigen receptor (CAR) to target cancer cells for adoptive immunotherapy.

## 2. Chemical and Physical Delivery Techniques

For gene expression, plasmid DNA has to cross three major obstacles: (i) the negatively charged plasma membrane, (ii) the cytoplasmic microskeleton environment, and, (iii) ultimately, the nuclear envelope, in order to reach the transcription machinery. The fact that plasmid DNA is a large hydrophilic polyanion represents a strong obstacle to the crossing of the hydrophobic plasma membrane. In the attempt to increase the transfection efficiency for enhancing the gene expression, nucleic acids have been complexed with chemical vectors such as cationic polymers, nanoparticles or liposomes. They favor cell transfection by mediating plasmid entry by endocytosis and protect DNA from nuclease degradation, although they do not allow for a total DNA release [7].

During the COVID-19 pandemic, lipid nanoparticles (LNPs) have been loaded with mRNA sequence encoding the Coronavirus spike protein. LNPs are traditionally composed of four components: an ionizable cationic lipid, an amphipathic phospholipid, cholesterol, and a polyethylene glycol-linked lipid (PEG-lipid). Combining LNPs with nucleic acids allows their protection against nucleases, does not preclude repeated administrations and has been shown to mainly target the liver after intravenous administration. Interestingly, without modifying the four-component ratios which have been optimized for an efficient nucleic acid encapsulation and endosomal escape, a concept referred as selective organ specificity (SORT) was developed to govern the biological fate [8]. By adding a fifth component, such as a quaternary aminolipid in an increasing amount, the nucleic acid delivery was relocated from the liver to the spleen in the majority, and finally to the lungs [8,9]. Notably, Li et al. [10] recently synthesized and evaluated an ionizable lipid, RCB-4-8, as a nanoparticle component for pulmonary mRNA delivery and gene editing in the lung. Other surface modification strategies involve galactosylation or mannosylation to target the nucleic acid delivery to hepatocytes and macrophages, respectively [11]. These few examples illustrate the fact that no unique formulation can be applied to all applications, which confers a certain level of complexity but offers an increased tissue or cell specificity.

Alternative non-viral techniques for gene delivery involve physical methods such as electrotranfer (also referred to as electrogene transfer, electroporation or nucleofection), sonoporation, and hydrodynamic gene transfer. Electrotransfer utilizes an external pulsed electric field for creating transient pores of a critical size within the lipid bilayers, thus modifying the cell membrane permeability and allowing DNA to migrate into the cell. In addition, because of the polyanionic nature of DNA, the electric field is inducing the DNA electrophoretic mobility, which plays a critical role for the efficiency of the plasmid DNA intracellular uptake [12]. This physical procedure is widely used for the in vitro transfection of a very large variety of cells, and also increasingly for in vivo organ transfection, most widely in skeletal muscle for prolonged transgene expression. The in vivo plasmid electrotransfer has also proved its efficacy in skin tissue, mainly targeting keratinocytes and antigen-presenting cells. Both muscle and skin electrotransfer are at a preclinical and clinical stage and were used to mediate the genetic immunization [13,14,15]. For hard-to transfect primary cells, such as hematopoietic stem cells (HSC) and T cells, nucleofection that combined different electrical voltages and conductive buffers enables plasmid DNA to cross the nuclear envelope and enter the nucleus [16].

The hydrodynamic gene transfer consists of injecting a large volume of DNA solution (8–10% of the rodent body weight) in less than 10 s via the tail vein, which creates a hydrodynamic pressure that expands the liver and increases the permeability of the capillary endothelium, thus allowing for gene delivery to adjacent parenchymal cells. The short and elevated hepatic pressure ensures a transfection of up to 40% of hepatocytes throughout the organ in small rodents and causes only transient damage [17,18]. This technique is largely applied in small rodents to compare liver-specific promoters in hepatocytes, but still awaits further developments for its translation in larger animals and humans. In dogs or non-human primates, the current procedure involves a liver lobe-specific, computer-controlled and image-guided plasmid hydrodynamic gene delivery [19,20].

Pore formation and cell membrane permeability can also be generated by the laser-assisted transfection [21] or ultrasound (also called sonoporation) that induced the growth and collapse of microbubbles [22,23].

Once in the cytoplasm, naked DNA complexed with cellular proteins is transported toward the nucleus along the cytoskeleton. The last but significant obstacle to gene expression is the nuclear envelope that only allows for a passive diffusion of molecules smaller than 40 kDa (diameter around 39 nm) through the nuclear pore complexes [24,25].

The size of a eukaryotic expression cassette, composed of one or several coding sequence(s) (as observed for the CAR T cells production; see Section 8), a promoter and polyadenylation sequences, is highly variable but generally within the range of 2.5–5.0-kb (i.e., 1.65–3.3 MDa). The mechanisms by which plasmid DNA enters the nucleus still contain grey areas, mainly in quiescent cells. In dividing cells, the disassembly of the nuclear envelope occurring during mitosis is considered to be a major factor for the nuclear plasmid localization and subsequent superior transgene expression.

In summary, the selection of the optimal transfection procedure is governed by a variety of parameters that include the characteristics of the cell type to be transfected, the cost of transfection reagents, and various outputs such as reproducibility, efficiency and minimal toxicity.

## 3. Rationale for Plasmid Optimization

For non-viral approaches, eukaryotic transgene expression vectors are mostly plasmids or constructs derived from plasmids. They present the advantages of being easily manufactured and produced using Good Manufacturing Practices (GMP) in a cost-effective manner. After the purification process, plasmids are either used ex vivo or in vivo in gene therapy protocols (complexed to the chemical vector or as naked DNA), or used as ancillary material for the production of viral particles or as templates for mRNA synthesis during the in vitro transcription process.

First-generation plasmids used for gene therapy and DNA vaccines contain two main domains harboring sequences of prokaryotic and eukaryotic origins. The eukaryotic expression cassette (EEC) is composed of a DNA sequence encoding one or several proteins placed under the control of a regulatory element, which is either a ubiquitous or a tissue-specific promoter, and a polyadenylation signal which is required for mRNA export and stability. The bacterial backbone contains an origin of the replication required for the plasmid amplification in an *Escherichia coli* strain and an antibiotic resistance marker allowing the selection of plasmid-containing bacteria.

Over the last twenty years, major avenues have been simultaneously explored to increase the yield of plasmid production, prolong transgene expression, and reduce cytotoxicity and/or inflammatory properties presumably associated to CpG sequences from prokaryotic origins [26]. Indeed, smaller non-viral transgene expression vectors display favorable properties, which include a higher transfection efficiency in vitro, ex vivo and in vivo as compared to first-generation plasmids consequently resulting in a higher transgene expression level [27,28,29]. The proposed mechanism is a facilitated journey for smaller gene carriers through cytoplasmic obstacles to the nucleus, and a higher resistance to shear stress generated by physical delivery techniques. Notably, using several DNA vectors with a size ranging from 281 to 5302-bp, Catanese et al. [30] established that shearing is strongly correlated with the DNA length when nebulization is used to create aerosol to deliver DNA drugs through the nose and mouth for the treatment of lung diseases. Furthermore, their study also established that at a fixed DNA length, the supercoiled DNA is considerably more resistant to shearing forces than linear DNA vectors.

New generation gene vectors have been developed to increase plasmid production yield and to meet the requirements of regulatory agencies that recommend avoiding the use of antibiotics during the manufacturing process, whenever possible, and thereby the removal of antibiotic resistance selection markers from the plasmid backbone [31,32]. The deletion of unwanted and redundant sequences presents several advantages, such as (i) not only a reduction in plasmid sizes favoring a decrease in metabolic burden for the bacterial host during the replication process of the high copy number of plasmids, but also (ii) the elimination of both the transcription and translation of the antibiotic resistance gene, as well as (iii) the decrease in the content of immunoinflammatory CpG sequences [26,33].

**Safety consideration concerning the use of classical plasmids as ancillary products for AAV production.** As stated above, AAV use for preclinical and clinical gene therapy has significantly developed over the last few years. The production of recombinant AAV (rAAV) is accomplished by co-transfecting a producer cell line, such as human embryonic kidney (HEK293T) cells, a procedure which generally needs three plasmids. The first one contains the EEC flanked by 145-bp-inverted terminal repeats (ITRs). The viral replication (Rep), structural (Cap) packaging and assembly activating proteins are encoded by a second plasmid, thus replacing the naturally AAV DNA sequence. Additionally, the generation of rAAV requires helper functions that are provided by a third plasmid containing adenovirus genes or relies on the infection of packaging cell lines with unrelated viruses such as the adenovirus. Of concern, Chadeuf et al. [34] first reported that encapsidated DNA into rAAV also contains prokaryotic sequences that represent 1–8% of the total vector genomes for single-stranded AAV vectors (ssAAV). For self-complementary AAV vectors (scAAV) containing both sense and antisense transgene sequences, encapsidated prokaryotic sequences represent up to 26.1% [35]. Most unwanted prokaryotic sequences originate from the plasmid carrying the EEC flanked by ITRs. Posing more of a concern, the functional ampicillin resistance gene was transferred and persisted in vivo in different tissues, conferring the risk of an integration into the host genome and expression under the control of a eukaryotic promoter [34]. Notably, rAAV vectors with higher purities and increased transduction efficiencies were reached, even for scAAV, when standard packaging plasmids were replaced by minicircle gene vectors (see Section 4 for a description of minicircles) [35].

Chadeuf et al. [34] hypothesized that the packaging of prokaryotic backbone sequences could partly result from an unspecific binding of the AAV Rep68 protein, a key viral protein required for the terminal resolution process by which the virus maintains its genomic ends, to heterologous Rep-binding site (RBS) sequences that are still present in current plasmids [34,36]. It can be suggested that the removal of such RBS sequences from first-generation plasmids or the use of RBS-free and antibiotic-free gene vectors could definitely increase the purity of rAAV vectors.

**Figure 1:**
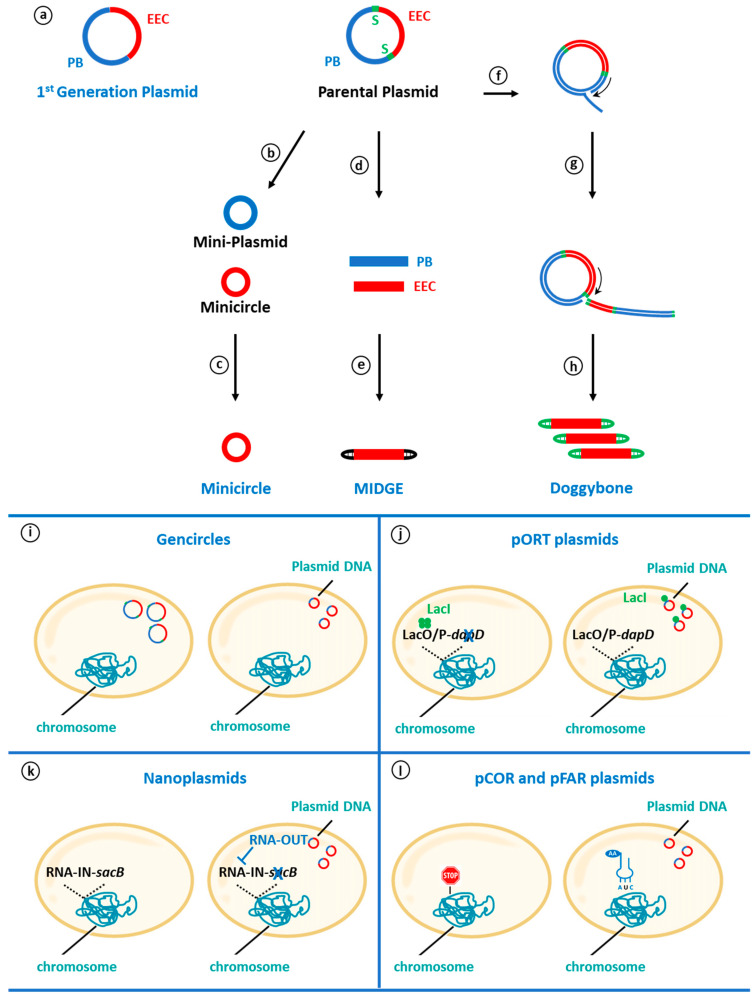
Strategies for the production of antibiotic-free gene vectors. First-generation plasmid contains a eukaryotic expression cassette (EEC) and a plasmid backbone (PB) composed of an origin of replication and an antibiotic resistance marker required for plasmid replication and maintenance in plasmid-containing bacteria, respectively (**a**). To produce antibiotic-free plasmids devoid of sequences of prokaryotic origin (Section 4), a parental plasmid is first produced in *E. coli*. For the minicircle production, the EEC is bordered by specific sites (S) to which a recombinase binds to mediate the intramolecular recombination (**b**) that generates two new molecules: a miniplasmid and a minicircle that is further purified (**c**). The production of MIDGE vectors involves the cleavage of the parental plasmid at specific restriction sites (S) by restriction enzymes to generate the linearized PB and EEC fragments (**d**). To the latter, hairpin oligonucleotides are ligated to covalently close the linear MIDGE vector molecule (**e**). To produce Doggybone^TM^ vectors, the parental plasmid, containing an EEC flanked with telomeric ends (TelL and Tel-R (S)) (**f**), is used as a template in a rolling circle amplification reaction that produces concatemers (**g**). The digestion of these latter with the TelN protelomerase at the Tel sites generates monomers with covalently closed ends (**h**) (adapted from Shafaati et al. [37]). Antibiotic-free gene vectors containing a reduced amount of sequences of prokaryotic origin (Section 5 and Section 6), comprise Gencircles^TM^, pORT, nano-, pCOR and pFAR plasmids. Gencircles^TM^ are produced from a parental plasmid carrying a kanamycin resistance marker, flanked by recombination sites, a R6K origin of replication and an EEC. The in vivo recombination eliminates the antibiotic resistance maker (**i**). The production of the pORT plasmids requires a modified strain genetically modified to contain an essential *dapD* gene regulated by the operator/promoter of the lactose operon. The bacterial survival is conditioned by the addition of an inducer in the growth medium or the presence of a pORT plasmid containing *lacO* sequences that titrate out the LacI repressor from the LacO/P promoter (**j**). The bacterial strain used to produce the nanoplasmids^TM^ contains a *sacB* gene inserted into the chromosome that encodes a levansucrase mediating toxicity in the presence of sucrose. The hybridization of the RNA-OUT sequence encoded by the nanoplasmids with the RNA-IN present at 5′-end of the *sacB* gene inhibits its expression resulting in bacterial survival (**k**). Finally, the production of the pCOR and pFAR plasmids requires an *E. coli* mutant containing an amber non-sense mutation in the *argE* or *thyA* gene, respectively. The resulting arginine and thymidine auxotrophy can be corrected to prototrophy upon the introduction of the pCOR or pFAR plasmids that encode a suppressor t-RNA that restores the ORF of the essential genes (**l**).

To produce antibiotic-free plasmids, several alternative selection procedures have been elaborated upon and promoted the development of novel gene vectors that can be divided into two main classes. Gene vectors of the first group, which include MIDGE, Ministrings, DoggyBone or minicircles, are totally devoid of sequences of prokaryotic origin. The second group that comprises Gencircles, pORT, Nanoplasmids, pCOR and pFAR miniplasmids harbors an origin of replication and, in some cases, a short prokaryotic sequence to select plasmid-containing bacteria (see Figure 1). The following sections describe their main characteristics, provide a few examples of pre-clinical tests obtained in the field of gene and cell therapies to mostly focus on the description of completed and ongoing clinical trials when available (see Table 1). An emphasis has been made on the pFAR4 gene vector to describe our recent data (see Section 6).

## 4. Non-Viral Expression Vectors Totally Devoid of Sequences of Prokaryotic Origin

**Minicircles** have been the first DNA molecule devoid of the antibiotic resistance gene introduced for gene therapy [38]. They are small, circular DNA molecules generated in *E. coli* from a parental plasmid that contains an EEC flanked by DNA sequences recognized by a recombinase enzyme (Figure 1c). After plasmid propagation, the induction of the in cellulo intramolecular recombination process generates two novel DNA circular molecules: the minimalistic EEC-carrying minicircle and a miniplasmid with the remaining part of the parental plasmid.

To generate the intramolecular recombination process, different tyrosine or serine recombinases have been evaluated. Tyrosine recombinases included the integrase of bacteriophage lambda [28,38], the Cre recombinase from bacteriophage P1 (at *lox* sites) [39], and the FLP recombinase of the yeast plasmid 2-μm circle (at FRT sites) [40]. These recombinases mediate a bidirectional and reversible recombination that results in several multimer structures due to intramolecular and intermolecular recombination. In contrario, the integrase of bacteriophage Phic31, a serine recombinase, catalyzes a unidirectional recombination between non-identical *attB* and *attP* sites to generate two novel hybride *attL* and *attR* [41,42] without generating concatemers. The second example of a unidirectional recombination system comprises the ParA resolvase from the multimer resolution system (*mrs*) of the broad host range, plasmid RK2 or RP4 [43,44]. The ParA resolvase only mediates intra-molecular recombination events at a higher yield without generating multimers or other concatemer events [45].

To minimize the propagation of the miniplasmid that inherits the prokaryotic origin of replication, the recombination process will not occur at early stages of bacterial growth but will require the induction of the expression of the DNA recombinase by either a temperature shift [28,38,40] or by placing the enzyme production under the control of the arabinose expression system [39,41,42,43,44]. However, in spite of this procedure, minicircle preparations still contain parental and miniplasmid contaminants and need to be further purified. To this aim, several purification methods have been developed, such as an affinity chromatography based on either the triple-helix formation [46], a DNA/protein interaction or the enhanced triplex DNA (TriD) technology [47]. The affinity chromatography based on the DNA/protein interaction relies on the interaction between the repressor of the lactose operon, the LacI protein, bound to a solid support, and a direct repeat of modified lactose operator sites inserted into the minicircle sequence [44]. The Triple-helix affinity chromatography and TriD-mediated MC purification is based on the reversible formation of the triple-helical structure between a biotinylated single-stranded poly-pyrimidine oligonucleotide and a double-stranded poly-purine DNA region introduced within the minicircle sequence, which can subsequently be either released by a pH jump [46] or efficiently captured by streptavidin-coated beads and isolated by a magnet [47]. To facilitate the minicircle purification procedure, another approach involves the digestion of the parental and mini-plasmids using a restriction enzyme that cleaves, ex cellulo, one of two DNA strands, thus generating open circle molecules (as opposed to supercoiled minicircle molecules) [48,49]. Alternatively, the DNA cleavage of both strands induced in the host bacterial strain after the recombination event linearizes both the parental and miniplasmid contaminants, which are then rapidly degraded by endogenous exonucleases while leaving intact the minicircle vector to be further purified [42].

Since the first article published by Darquet et al. [38], dozens of studies have been published describing the use of minicircles as gene vectors. Only a few examples are cited with the sole intention to illustrate the diversity of applications. The transfection of embryonic stem-cell-derived neural stem cells (NSC) with a minicircle did not affect cell division kinetics, morphology or differentiation potential to astrocyte and neurons [50]. For a non-viral topical treatment of patients with Recessive Dystrophic Epidermolysis Bullosa (RDEB), a severe genetic skin disorder caused by mutations in the COL7A1 gene, RDEB skin cells were transfected with a minicircle expressing the large cDNA size of the COL7A1 gene (8.9-kb) to restore the expression and secretion of the structural protein-type-VII collagen (C7) at the dermal and epidermal junction [51]. The transfection of mesenchymal stem cells (MSCs) using a minicircle expressing the human angiopoietin 1 (ANGPT1) enhanced the MSC therapy by mediating a reduced pulmonary inflammation and lung permeability in lipopolysaccharide-induced acute lung injury (ALI) in mice [52]. The ex vivo transfection of bone marrow-derived MSC with a minicircle expressing the vascular endothelium growth factor (VEGF) has been performed to promote endothelial cell growth and survival for tissue repair in patients with peripheral artery disease (PAD) which is characterized by a blockage of the arteries and a decrease blood flow to the lower extremities [53]. Additionally, a minicircle expressing the transcription factors *OCT4*, *SOX2*, *NANOG* and *LIN28* was introduced into human adipose stromal cells to generate the human-induced pluripotent stem cells (hiPSC) towards a subsequent differentiation to other cell types for disease pathology investigation and regenerative medicine therapies [54]. In addition, like the pFAR4 miniplasmids, minicircles promote a prolonged transgene expression in the liver (see Section 6).

In 2020, the minicircle gene vector has been included in a phase I/IIa clinical trial to propose an alternative treatment for patients with multiple myeloma (MM), a rare hematological malignancy. The innovative immunotherapy approach aims at genetically modifying autologous white blood cells to express a chimeric antigen receptor (CAR; see Section 8) targeting SLAMF7 (Signaling Lymphocytic Activation Molecule Family 7), a surface protein expressed mainly on MM cells, to destroy the malignant cells (Table 1 #NCT04499339) [55].

Minimalistic, Immunologically Defined Gene Expression (MIDGEs), Ministrings and Doggybone DNAs (dbDNA^TM^, named after their schematic structure) are linear, covalently closed constructs that are only composed of a gene of interest (GOI) and the appropriate regulatory elements. They are generated from a purified parental plasmid that carries an EEC flanked by either two specific restriction sites for the former or two telomeric ends. 

To generate **MIDGE** vectors, plasmid DNA is digested with an appropriate restriction enzyme, and the relevant fragment is ligated to hairpin oligonucleotides to close the linear eukaryotic cassette, which is finally purified by anion-exchanged chromatography (Figure 1e) [56]. MIDGE vectors have been used for DNA-based therapies and vaccines against Feline Immunodeficiency Virus (FIV) using DNA sequence encoding domains of the *env* gene and the feline IL-12 cytokine [57,58]. In addition, an improved MIDGE vector that contains a nuclear localization sequence bound at one of the two ends, was designed to favor an increased expression of either the LACK antigen for protection against Leishmania [59], the hepatitis B virus surface antigen (HBsAg) [60,61], or a truncated, secreted form of bovine herpesvirus-1 (BHV-1) glycoprotein D (tgD) [62].

A phase I clinical trial aims at investigating the efficacy of the expression of the tumor necrosis factor (TNF-α) in cutaneous metastases of malignant melanoma after the intratumoral jet injection of the MIDGE gene carrier. The primary objective of this study is to establish a local treatment of metastases with a systemic effect (Table 1 #DRKS00005723). The second phase I/II study investigates the safety and efficacy of an intradermally administrated tumor vaccine (MGN1601) that consists of genetically modified allogeneic (Human) tumor cells for the expression of IL-7, GM-CSF, CD80 and CD154 in combination with the TLR-9 agonist dSLIM (**d**ouble **s**tem **l**oop **i**mmuno**m**odulator) in Patients With Advanced Renal Cell Carcinoma (RCC) (ASET Study). MGN1601 was reported to be well tolerated and safe with the potential to treat RCC patients (Table 1 #NCT01265368) [63].

The production of **Ministrings** exploits the *Yersinia enterocolitica* bacteriophage PY54-derived Tel/*pal* protelomerase system to generate a linear covalently closed vector from a plasmid backbone [64,65]. The EEC contains four copies of the SV40 enhancer (SV40E) that binds transcription factors, the nuclear localization sequence of which mediates the importation of the DNA-protein complex to the nucleus and enhanced transgene expression [64,65].

To facilitate the generation of covalently closed linear fragments, a PCR approach has been developed to generate **Doggybone**^TM^ (db) vectors (Figure 1h). Using plasmid DNA as a template and the high fidelity Phi29 polymerase, DNA concatemers are first produced in a rolling circle amplification (RCA) reaction [37]. The resulting DNA concatemers are digested by the TelN protelomerase, isolated from the *E. coli* prophage N15, which cuts the double-stranded DNA at the telomeric ends and subsequently generates covalently close ends with short hairpin loops [37]. The production of db vectors is reported to be five times faster than plasmid DNA as it does not require the production of a master cell bank. This novel approach is still in its infancy and has been assessed as an ancillary material for lentivirus production [66] and DNA vaccines to target the human immunodeficiency virus (HIV), influenza, Human Papillomavirus (HPV) or the SARS-CoV-2 spike protein [67]. Equivalent humoral and cellular responses to those obtained with plasmid DNAs have been reached. A db vector has also been paired with the *PB* transposon to engineered CAR-T cells [68] (see Section 8).

The Doggybones and other linear synthetic double-stranded linear DNA, covalently closed with single-strand hairpins at the 5′ and 3′ ends and produced by enzymatic processes for gene therapy applications, have been recently introduced; hpDNA™ or NeDNA™ are two other examples available commercially.

## 5. Plasmids Devoid of Antibiotic Resistance Marker and Containing Minimized Prokaryotic Sequences

Antibiotic-free plasmids of this group contain, in some cases, a short prokaryotic sequence to select plasmid-containing bacteria and an origin of replication either derived from pMB1 or R6K plasmids.

**Origin of replication used to propagate antibiotic-free non-viral gene vectors:** As extrachromosomal genetic elements that replicate autonomously in bacterial hosts, plasmid DNAs contain their own origin of replication. Most therapeutic plasmids contain a pMB1 or ColE1-derivatives (pUC-type) origin of replication that promotes a high plasmid copy number (500–700 copies) in an *E. coli* strain to favor a high-yield plasmid production. The regulation of the replication initiation is mediated through an RNA/RNA hybridization between the primer RNA and the antisense sequence [69]. Alternatively, plasmids may harbor a conditional R6K origin of the replication that requires the binding of the π protein to initiate the replication process. To reduce the plasmid size, the *pir* gene encoding the π protein is provided in *trans* by insertion into the bacterial chromosome. With the original *pir* gene, R6K-containing plasmids will be present at ~10–15 copies per cell. Mutagenesis of the *pir* gene results in an increase in the R6K replicon copy number, which was estimated to 400 copies per cell [70,71,72].

**GenCircles**^TM^ are generated in an *E. coli* strain from a kanamycin resistant parental plasmid that contains recombination sites bordering the R6K origin of the replication and gene of interest (GOI). In cellulo recombination events lead to the formation of two circular DNA molecules composed of (i) the sole kanamycin resistant marker that therefore does not replicate and (ii) the Gencircle composed of the R6K and GOI DNA sequences (Figure 1i). By virtue of the plasmid incompatibility, parental plasmids and GenCircles^TM^ cannot be maintained inside the same bacterial strain. More than 80% of *E. coli* cells were reported to contain pure GenCircles^TM^ that are used for plasmid production. Gencircles are small and circular DNA vectors with a 429-bp backbone. Their potency has been assessed as a transposon vector and CRISPR knock-in template, for lentivirus and AAV packaging (https://www.genscript.com/gencircle-double-stranded-dna.html (accessed on 20 December 2023)).

**pORT plasmids:** The Operator Repressor Titration (ORT) system exploits the features of the bacterial lactose operon (Figure 1j). The *E. coli* strain engineered to produce pORT plasmids harbors a modified copy of the *dapD* gene, the expression of which is under the control of the *lac* promoter/operator sequence to which the LacI repressor binds [73,74]. The DapD protein encodes the tetrahydrodipicolinate N-succinyltransferase, an enzyme playing a key role in the lysine/diaminopimelic acid (DAP) pathway involved in the cell wall biosynthesis. In the engineered *E. coli* strain, the conditional bacterial growth is mediated by the induction of the *lac* promoter by either galactose or isopropyl β-D-1-thiogalactopyranoside (IPTG) or in the presence of a multi-copy pORT plasmid that contains 20-bp short operator sequences that titrate out the LacI repressor to mediate the expression of the *dapD* essential gene [73,74]. The pORT plasmids have mostly been used to design DNA vaccines. Preclinical tests were performed with a pORT plasmid expressing either the LACK antigen for vaccination against canine leishmaniasis [75] or the interleukin-12 protein electrotransferred to mouse, canine and human melanoma cells to pave the way for a human clinical trial [76].

Two phase I clinical trials were initiated using a plasmid AMEP that encodes an Antiangiogenic MEtargidin Peptide derived from the disintegrin domain of human metargidin (also known as ADAM-15) which binds to α5β1 and αvβ3 integrins upregulated on endothelial cells during tumor angiogenesis. To assess the potency of the AMEP integrin antagonist and thereby antiangiogenic effect, the plasmid AMEP has been either injected into advanced solid tumors (Table 1 #NCT01045915) or into femoral muscle (Table 1 #NCT01664273) before being electrotransferred using a combination of one high voltage pulse, to favor cell permeabilization, followed by one low voltage pulse, to enable a DNA-membrane interaction. The intratumoral injection of plasmid AMEP into cutaneous metastatic melanoma did not reveal serious related adverse events. The measurement of transfected lesions suggest a stabilization reflecting a local anti-tumor efficacy that requires, however, it to be statistically confirmed with a higher number of patients [77]. The intramuscular injection of plasmid AMEP to patients with advanced solid tumors aimed at evaluating distant effects by blood circulation of the AMEP peptide. No objective responses to the otherwise well tolerated treatment were observed that could reflect either a below limit detection level of the AMEP protein, absence of the targeted integrins in the tumors or lack of systemic antitumor effectiveness [78].

**Plasmids produced using an RNA/RNA antisense interference procedure:** To construct and produce biosafe plasmids, two different approaches based on the RNA/RNA antisense interference have been developed.

The first one is derived from the ColE1 origin of replication that encodes two antisense RNAs, RNA-I and RNA-II, which are involved in the regulation of the plasmid copy number. In the engineered bacterial host, the RNA-I encoded by the plasmid origin of replication hybridizes with the RNA-II fused to the *tetR* repressor sequence to suppress its translation and its binding to the *tetO* promoter located upstream of the *murA* essential gene (which is involved in the first step of the bacterial cell wall), thus promoting bacterial growth [33,79]. These selection procedures only rely on the origin of replication and therefore precludes the need of additional prokaryotic sequences on the plasmid backbone, but are limited to plasmid vectors containing a ColE1-type origin of replication.

The second system is an adaptation of the mechanism which regulates the transposition event of the insertion sequence 10 (IS10) that encodes two complementary and divergently orientated RNAs, RNA-IN and RNA-OUT, which inhibit the expression of the downstream transposase gene to limit the IS10 copy number in a cell. The Nanoplasmid^TM^ technology exploits this process. The nanoplasmid vector backbone carries a R6K origin of replication and the 150-bp RNA-out coding sequence that hybridizes with an RNA-In-*sacB* construct inserted into the host chromosome (Figure 1k). The RNA-out-mediated-repression of *sacB*, which encodes a lethal levansucrase when growing bacteria in the presence of sucrose, abolishes cell death and allows for plasmid propagation [80,81]. Nanoplasmids have been employed in a variety of preclinical tests in the field of gene therapy, DNA vaccination and immunotherapy, which resulted in several clinical trials (Table 1).

The #NCT03288493 phase I/II clinical trial aims at targeting the B-cell maturation antigen (BCMA) in patients with Relapsed/Refractory Multiple Myeloma using P-BCMA-101 autologous T stem cell memory (Tscm) Chimeric Antigen Receptor T cells (see Section 8). The treatment was reported to be well tolerated, with minimal toxicity, and marked efficacy [82,83].

Three clinical trials (#NCT01707069; #NCT01966224; #NCT02146781) [84] aimed at investigating the safety and immunological responses of a DNA vaccine encoding the CryJ2 allergen from the Japanase Red Cedar pollen, which affects one third of Japanese people, as well as the Lysosomal Associated Membrane Protein 1 (LAMP-1) that enhances both cellular and humoral responses in vaccinated animals. The DNA vaccine administrated either intramuscularly or via an intradermal route using a Biojector device was well tolerated without generating serious safety issues. In allergic patients, potential immunological therapeutic effects were observed. As stated by Su et al. [84], these promising results need to be confirmed with a higher number of patients and an additional biomarker measurement.

The aim of these phase I and II clinical trials (#NCT02301754, #NCT03265717, #NCT04515043) [85] is to evaluate the potency of INVAC-1, developed for cancer therapy (hematologic malignancies and solid tumors) by targeting the human telomerase reverse transcriptase (hTERT). The hTERT protein, the catalytic subunit of the telomerase complex, is highly expressed in more than 85% of human tumors where it is playing a role in the unlimited proliferative capacity of cancer cells, while displaying little or no expression in normal somatic cells. The studies investigated the potency of INVAC-1, a DNA plasmid encoding an enzymatically inactive hTERT protein fused to a ubiquitin moiety to favor its degradation and peptide presentation, thus enhancing the naturally occurring hTERT-specific immune responses’ induction. The intradermal injection of INVAC-1 followed by either an electroporation or needle-free injection system was safe and well tolerated and elicited both hTERT-specific CD4 and CD8 T-cell immune responses in patients with relapsed or refractory solid tumors. The majority of patients (58%) experienced disease stabilization during the treatment period and beyond.

In the field of eye diseases, phase I/II (#NCT03308045) [86] and a phase II (#NCT04207983) [87] clinical trials aim at assessing a novel therapeutic approach for patients with non-infectious uveitis, an immune-mediated inflammatory disease that affects the uvea which can result in severely reduced vision or blindness. Administration and electrotransfer, in the ciliary muscle, of a plasmid expressing a secreted anti-Tumor necrosis factor α (TNFα) fusion protein was designed to neutralize the TNFα cytokine that plays a key role in the mediation of intraocular inflammation. Adverse events following the treatment were reported to be mild to moderate in severity and resolved by the end of the study. Clinically significant improvements were reported.

The #NCT04752722 phase I/II study investigates the safety and efficacy of EG-70, an immune-oncology gene therapy for patients with non-muscle Invasive Bladder Cancer (NMIBC). The intravesical administration of EG-70 in the bladder, a nanoparticule formulation containing a nanoplasmid encoding both the retinoic acid-inducible gene I (RIG-I) agonist [88] and IL-12, aims at locally inducing a potent immune response in the tumor environment and avoiding systemic toxicities. The treatment did not generate dose-related adverse events. Out of the 19 patients treated, 18 completed one cycle of EG-70 treatment and 67% achieved a complete response [89].

The phase I/II clinical study (#NCT04591184) aims at investigating the safety, tolerability and immunogenicity of Covigenix VAX-001/-1b after two intramuscular injections in healthy adults. The DNA vaccine, encapsulated in proteolipid vehicles, expresses the full-length SARS-CoV-2 spike protein combined with CpG motifs and the RIG-I agonist, two genetic adjuvants that stimulate the immune response.

Phase I (#ACTRN12613000831785) and phase I/IIa (ACTRN1261500009457**2**) [90] clinical trials evaluated a DNA-based immunotherapy to genital herpes upon the intradermal delivery of COR-1 which is a 1:1 mixture of two plasmids: (i) one encodes a codon-optimized full-length envelope glycoprotein D of the herpes simplex virus type 2 (HSV-2) (gD2) and (ii) the other encodes a truncated version of gD2 fused to an ubiquitin sequence for the induction of a CD8^+^ T cell response after protein processing in the proteasome and presentation to the Major Histocompatibilty Complex (MHC) of class I. Both in healthy volunteers and HSV-2 positive patients, the injection of COR-1 was safe and well tolerated and did not induce serious adverse events. The administration of COR-1 in HSV-2 positive patients revealed trends in the reduction in viral shedding and in the induction of antigen-specific cellular and humoral immune responses in peripheral blood. This study awaits a further optimization of the vaccine to improve immunogenicity and clinical efficacy as well as the inclusion of a higher number of patients to detect a significance in changes in comparison with the placebo group.

## 6. Plasmids Devoid of Antibiotic Resistance Gene and Using a Suppressor tRNA as Selection Marker

The pCOR (Conditional Origin of Replication) and pFAR (Free of Antibiotic Resistance) miniplasmids contain a R6K and pUC-type origin of replication, respectively, and a suppressor t-RNA sequence expressed from prokaryotic regulatory sequences [71,91]. The suppressor t-RNAs encoded by the pFAR and pCOR plasmids restores the open reading frames (ORF) interrupted by an amber nonsense mutation introduced into a chromosomal essential gene (Figure 1l).

**pCOR plasmids.** The propagation of pCOR plasmids relies on the restoration of the *argE* ORF that encodes an arginosuccinate synthetase enzyme which is essential for the arginine synthesis. In selective media devoid of arginine or protein sources, the arginine auxotrophy of the *E. coli* mutant can only be corrected to prototrophy after the introduction of the pCOR plasmid [71,72].

The aim of the phase III clinical trial (Table 1 #NCT00566657) was to investigate the statistical significance of treating patients with critical limb ischemia, which have a high rate of amputation and mortality, with a pCOR plasmid expressing the Fibroblast growth factor 1 (FGF1). FGF1 activates the migration, proliferation and differentiation of endothelial cells, promoting new blood vessel formation that was hypothesized to improve amputation-free survival. Patients (n:525) with critical limb ischemia from 171 sites in 30 countries were included. They were randomly assigned to either the therapeutic treatment (eight intramuscular injections of FGF1-expressing pCOR) or a matching placebo. Although no clear adverse events were observed with the tested plasmid, the clinical outcome did not statistically differ between both groups, with major amputation or death occurring in 37% patients of the active group (96 out 259) and 33% patients in the placebo group (86/266) [92].

**pFAR plasmids**. The pFAR plasmids are produced from an *E. coli* strain containing an amber nonsense mutation introduced into the essential *thyA* gene of the MG1655 strain, the genome sequence of which has been fully determined [91]. The ThyA protein catalyzes the conversion of deoxyuridine monophosphate (dUMP) to deoxythymidine monophosphate (dTMP), a precursor required for DNA synthesis. The thymidine auxotrophy brought about by the lethal amber mutation can be overcome by adding thymidine in the bacterial growth medium to maintain the non-transformed strain. Inside the bacterial cells, thymidine is converted into dTMP via an alternative metabolic pathway involving the thymidine kinase enzyme. In thymidine-free selective media, prototrophic bacterial growth occurs upon the restoration of the *thyA* open reading frame mediated by the expression of the suppressor t-RNA gene delivered by the pFAR4 gene vector (Figure 1l).

Compared to the pCOR system, the main advantage of the pFAR technology resides in the possibility of using proteins-containing media, favoring a higher production yield. A nutrient rich-medium based on the LB Broth Lennox formulation has been successfully used for the plasmid production of research and clinical grade in shaker flasks and fermenter cultures. This medium, which is manufactured from animal-free ingredients, is composed of sodium chloride, yeast extract and soy hydrolysate that replaces the tryptone present in the initial formula. In parallel, a chemically defined medium has been identified to optimize the plasmid production process in fermenter cultures. To date, three clinical lots of pFAR plasmids have been produced for two phase I/II clinical trials (see below) [93].

The potency of the pFAR4 gene vector has been first assessed using the luciferase reporter gene and electrotransfer for plasmid delivery in mouse muscle, skin and transplanted tumor cells. In a longitudinal study using a charge-coupled device (CCD) camera, similar luciferase activities were recorded for at least two months in muscle transfected with the luciferase-encoded pFAR4 or the control plasmid expressing an identical expression cassette [91]. In comparison with a control plasmid bearing a kanamycin resistance gene, an almost one-log higher luciferase activity was reached after the transfection of transplanted tumor cells with the pFAR4-LUC plasmid. Similarly, a two-log increase and longer transgene expression was observed after the electrotransfer of the pFAR4 miniplasmid into skin. The reduced size of the pFAR4 gene vector promoted increased transgene levels compared to vector controls in all animal or human cells transfected using either cationic lipid, nucleofection, electroporation, and hydrodynamic delivery (see following section) [94,95,96,97,98,99,100,101].

**pFAR and minicircles both promote a sustained transgene expression in the liver.** The potency of non-viral gene vectors, including minicircles and pFAR4 plasmids, have been evaluated in hepatocytes, using a tail vein hydrodynamic delivery (see Section 2). The liver is a key targeted organ in which more than 66% of the human proteins are expressed, thus controlling around 500 functions that play a major role in various and essential biological processes, such as blood coagulation, metabolism, detoxification or immunity [102]. Furthermore, proteins secreted by the liver are properly processed and glycosylated, which represent relevant posttranslational modifications for the treatment of monogenic disorders of a hepatic origin and other pathologies such as lysosomal storage diseases or bleeding disorders.

The lifetime treatment of patients requires a long-lasting production of therapeutic proteins that are largely dependent on two main genetic elements: the bacterial plasmid backbone and the promoter driving the transgene expression. With the exception of the ubiquitin promoter that drives the sustained expression in hepatocytes, other ubiquitous promoters are subjected to silencing [99,103]. To overcome this effect, several liver-specific promoters have been identified such as a 415-bp DNA sequence derived from the human α-1 antitrypsin (hAAT) gene that codes the most abundant serine protease inhibitor in human plasma, which can be optionally coupled to the apolipoprotein E locus control region (HCR) [104,105]. In the liver, the group of M. Kay first recognized the additional key influence of the vector backbone feature for a sustained transgene expression. Indeed, despite the fact that plasmid DNAs are still present in the transfected hepatocytes, first-generation plasmids are subjected to a fast transgene silencing within two weeks post-delivery [41,100]. By comparing the transgene expression level after delivery of the EEC either as a linearized DNA fragment, a minicircle DNA molecule, or by plasmids of different sizes, it was established that transgene silencing is mediated by (i) a covalent linkage between the prokaryotic DNA sequences and the EEC [41,106]; (ii) the size of the plasmid backbone that should not exceed 1-kb [107]; and (iii) the GC composition of the plasmid backbone [108].

Although the underlying mechanisms involved in transgene silencing still need to be fully deciphered, several hypotheses have been put forward based on the following observations. Chromatin immunoprecipitation assays (ChIP) coupled with quantitative PCR (qPCR) highlighted an enrichment (more than five-fold) of the H3K27me3 heterochromatin mark in the 5′-end of EEC carried by antibiotic-resistant plasmids [100,103,109]. The tri-methylation state of the histone H3 protein characterizes a highly condensed chromatin form, which reduces access of the transcriptional machinery complex to the promoter region. In a marked contrast, minicircles and pFAR miniplasmids appear to remain in an open state, loosely packed and transcriptionally active chromatin form. Both the small size of the pFAR4 plasmid backbone (less than 1-kb) combined with a low GC value (42.9%) and AT-rich regions most probably favor the exclusion of nucleosomes, thus preventing transgene expression silencing [110]. In summary, it has been hypothesized that highly GC-enriched regions, such as prokaryotic sequences, favor heterochromatin formation that spread out to adjacent regions and EEC as observed when inserting an *E. coli* GC-rich sequence into the genome of embryonic stem cells (ESCs) [111]. Thus, antibiotic-free gene vectors, such as pFAR4 which contains a small plasmid backbone, offer an attractive solution to the transgene silencing occurring in the liver.

**Towards a cost-effective treatment for neovascular “wet” age-related macular degeneration (nAMD):** The superior potency of the pFAR4 plasmids in human retinal and iris pigment epithelial (RPE and IPE) cells led to their selection for a future phase I/II clinical trial aiming at proposing an alternative cell/gene advanced personalized biotherapy for nAMD. The therapeutic strategy of the Horizon 2020-supported TargetAMD consortium (www.TargetAMD.eu, accessed on 20 December 2023, coordinated by Pr. G. Thumann) is based on an ex vivo electroporation of autologous IPE cells, using two pFAR4 plasmids. The first plasmid expresses the pigment epithelium-derived factor (PEDF), a potent anti-angiogenic factor. The second plasmid encodes the SB100X transposase to mediate a transgene integration into the host chromosome (see the following Section 7 on transposons). The one hour procedure consists of transfecting IPE cells by electroporation and re-implanting the genetically modified cells subretinally into the patient’s eye.

A battery of preclinical assays has been performed [94,95,96,97,98,112], including in vitro IPE cells’ transfection, a quantification of the secreted PEDF level and the assessment of transgene integration sites which were, as expected with the *SB* transposon system, close to random (see Section 7 below on transposons). Furthermore, the transplantation of the genetically modified cells to the subretinal space of a rat model significantly reduced the laser-induced choroidal neovascularization and did not generate any change in rabbit organ morphologies (Biodistribution studies). Thus, on animal models, the increased PEDF local production counteracts the neovascularization effect mediated by the vascular endothelial growth factor (VEGF), which is overexpressed in nAMD.

In comparison with current treatments that mostly involve monthly intravitreal injections of VEGF inhibitors, such as antibodies, recombinant soluble receptors or “VEGF trap”, which could generate side effects, and suffer from insufficient patient compliance, the TargetAMD innovative procedure will be unique and also aims at curing patients not responding to existing treatments. Furthermore, during the TargetAMD program, it was estimated that recurrent injections of anti-VEGF monoclonal antibodies cost 25–30,000 euros/patient/year (only for the drug) while the proposed alternative treatment of one shot is expected to have a price of ~3500 euros/patient.

**pFAR4 gene vector human clinical trial for hearing disorders:** In August 2020, a pFAR4 plasmid encoding two neurotrophic factors, the brain-derived neurotrophic factor (BDNF) and neurotrophin-3 (NT3) proteins, entered a phase I/II clinical trial directed by Prof. G. Housley (UNSW, Sydney) (www.CINGT.info (accessed on 20 December 2023); Table 1 # ACTRN12618001556235). The first delivery of one of our plasmids in humans aimed to improve the hearing performance in patients with inherited or acquired deafness by coupling a cochlear implant and a gene therapy approach.

To date, depending on the degree of disability, patients with hearing disorders can benefit from various medical devices ranging from conventional hearing aids, which amplify sound, to cochlear implants. The latter are complex electronic devices that capture and deliver sound signals to the auditory nerve. Thus, cochlear implants bypass damaged portions of the ear. However, in patients with profound hearing disability, sensory hair cells that help to convert acoustic vibrations into nerve impulses (a process that allows one to hear), are absent. The causes can result from either a genetic defect, aging or external factors such as ototoxic chemicals, infections or high noise exposure. The definite loss of these cells generates the retraction of spiral ganglion neurites from the organ of Corti (also called spiral organ), resulting in a neural gap between the cochlear implant electrode and the auditory nerve fibers. Consequently, the sound quality perceived by the cochlear implant recipients is not optimal and can be considered metallic or atonal, and thereby results in a limited appreciation of music and tonal languages such as Mandarin or Cantonese.

The innovative approach proposed by Prof. G. Housley’s team (University of South Wales, UNSW, Sydney, Australia) uses a novel electric field focusing technology (BaDGE^®^) to electrotransfect the mesenchymal cells lining the cochlear perilymphatic compartment with a pFAR plasmid encoding the BDNF and NT3 neurotrophic factors. The rationale is that these neurotrophic factors stimulate the oriented regeneration of the peripheral neurites of the spiral ganglion neurons to close the neural gap existing between the cochlear implant and the auditory nerve fibers in deaf patients. Notably, preclinical tests including confocal imaging revealed that GFP-encoded pFAR4 was restricted to mesenchymal cells lining both scala tympani and scala vestibuli in the basal turn, in close proximity to the electrode array, even though all four turns of the guinea pig cochlea had been perfused [113]. In deafened guinea pigs, the secretion of neurotrophic proteins by transfected mesenchymal cells stimulated the regeneration of spiral ganglion neurites, which had been atrophied due to an ototoxic treatment. In addition, the neural remodeling restored hearing, as evidenced by electrically evoked auditory brainstem responses in animal models possessing destroyed hair cells [114].

The purpose of the ongoing phase I-II clinical trial is to evaluate the safety and efficacy of the combined neurotrophin gene therapy in conjunction with the cochlear implant, in comparison with patients that have the cochlear implant (control group) exclusively. No adverse events of concern linked to the investigational device, including the control of the DNA delivery, have been detected in patients of the gene therapy group. Furthermore, the DNA solution and electrotransfer were very well tolerated, demonstrating that the treatment has met the primary objective of the phase I study. A description of the ongoing clinical trial and patient experience for the first seven patients can be visualized following this link to the Australian TV news (https://www.facebook.com/watch/?v=3341989752711664 (accessed on 20 December 2023)). Final outcomes of the clinical trial await the 12 months follow-up of the last enrolled patients from the gene therapy and control groups. The launch of this study is the result of a fruitful collaboration initiated in 2015 between our research group and the University of South Wales team led by Prof. Housley, in partnership with Cochlear Ltd., Sydney-based co-investigators at NextSense, the University of Sydney and the Macquarie University Hearing Hub.

Hearing loss represents a major health and economic issue, as it has considerable effects on the individual quality of life with a major impact on communication, speech, cognition, education and employment, potentially generating social isolation and depression. To date, the World Health Organisation (WHO) estimates that over 5% of the global population (more than 432 million adults and 34 million children) suffer from hearing loss. With an increasing number and aging population, this proportion is expected to increase to ~10% of the world’s population, representing 700 million people by 2050 (https://www.who.int/news-room/fact-sheets/detail/deafness-and-hearing-loss (accessed on 20 December 2023)). Improving cochlear implant performances thus definitely constitutes, in the long run, a return of investment for both patients and health care systems.

## 7. Transgene Chromosomal Integration Using Non-Viral Techniques

Chemical and physical delivery methods mediate the entry of non-viral gene vectors that remain extrachromosomal. Plasmid integration studies performed after intramuscular injection and electroporation did detect independent insertion events which can be considered as negligible, as they occurred at a frequency that is below the spontaneous mutation rate [115,116]. Although the episomal nature of non-viral gene vectors has been considered as a valuable feature to avoid genotoxicity potentially mediated by retro- and lentiviruses’ integration into the host genome, it can also be observed as a limitation when the division of transfected eukaryotic cells leads to the dilution of non-replicating plasmids. The pairing of non-viral gene vectors with integrating transposable elements (TEs) has recently been assessed to bypass this limiting feature.

**Figure 2:**
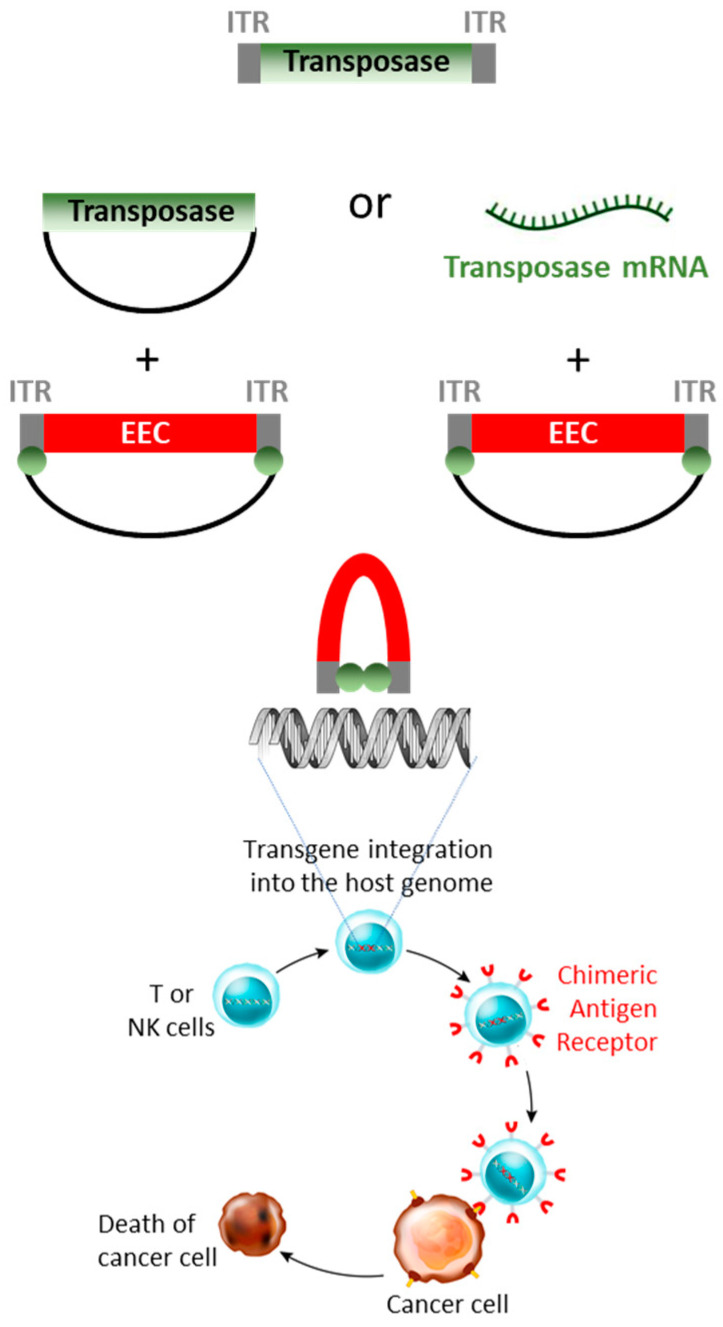
Transposase and DNA transposons mediate transgene integration into the host genome circumventing plasmid DNA dilution and loss of the therapeutic gene in dividing eukaryotic cells. Naturally, DNA transposons contain a transposase gene flanked by two inverted terminal repeats (ITRs). For gene therapy applications, eukaryotic cells are transfected with a plasmid carrying the EEC inserted between the two ITRs. The transposase is encoded either by a second plasmid or an mRNA and thus added in trans. Upon expression, the transposase binds to the ITRs, excises the transposon from the plasmid involving the formation of a synaptic complex, and finally inserts the EEC DNA into the host genome via a cut and paste mechanism. The transfection of T or NK cells with this approach generates genetically modified cells that stably express a chimeric antigen receptor (CAR) recognizing antigens present on the surface of cancer cells, and thereby mediates the death of the targeted cancer cells.

First identified in Maize by Barbara McClintock in the 1940–1950 decades [117], TEs are mobile genetic elements that move from one locus to another using either an RNA or DNA intermediate. The propagation of retrotransposons occurs via a copy-and-paste mechanism that involves the transcription of the TE, the reverse transcription of the generated RNA into a cDNA that integrates into a second locus. Class II natural DNA transposons encode a transposase protein that binds to the inverted terminal repeats (ITRs), delimiting the transposon ends (Figure 2). The excision and integration activity of the transposase protein promotes the transposon mobilization via a cut-and-paste mechanism. In humans, TEs represent around 45% of the genome sequence and were originally regarded as mediators of genetic variability, as they are a main source of chromosomal recombination and mutation, until their overall transposition activities declined markedly [118]. In less than two decades, DNA transposons have been redesigned and integrated in the genome engineering field and have become key additional components of the non-viral Gene Therapy Toolkit.

By inserting either a unique or a multicistronic expression cassette between specific transposon ITRs, the properties of DNA transposon can be exploited to mediate the transgene integration into the genome of host cells. To this end, cells are transfected in vitro, using a two-component system composed of a plasmid carrying a transposon encoding the sequence of interest and a separate genetic element (plasmid or mRNA) encoding the transposase. Currently, three DNA transposons are mostly used for gene therapy applications: *SB*, *PB* and *TcBuster*, which are representative of the *Tc1/mariner*, *PB* and *hAT* superfamilies, respectively.

***SB*** **DNA transposons.** The SB transposase was awakened by constructing a consensus DNA sequence derived from inactive Salmonid genomes and eliminating the mutations affecting the protein open-reading frame, nuclear localization signal, DNA binding and integration activities [119]. Subsequently, a hyperactive transposase (SB100X) that promotes a ~100-fold improvement of integration efficiency compared to the original SB transposase was obtained by DNA shuffling and found to mediate the DNA transposition in a variety of vertebrates including mice and human cells [120]. During the transposition process, the *SB*-mediated transposon excision leaves behind a small and characteristic CAG footprint mediated by DNA repair [121]. The *SB* transposon predominantly targets TA dinucleotides (which become duplicated at the integration site), and preferably at an 8-bp palindromic ATATATAT repeat sequence [122]. The *SB* transposition efficiency is negatively affected by two different factors: (i) an excess of transposase that mediates a process called the overproduction inhibition (OPI) [123]; (ii) a size increase in the transgene to be delivered into host genomes [124]. Notably, the distribution of *SB* transposon insertions into the host genome shows a close-to-random integration profile in mammalian genomes, with a minor bias towards transcription units and their upstream regulatory sequences [125]. Finally, recently identified SB100X variants (K248R and H187V) redirect integration away from genes and transcriptional regulatory elements, thus further lowering the risk of insertional mutagenesis [126].

***PB*** **DNA transposons.** The second DNA transposon of relevance for gene and cell therapy application is the *PB* transposon system, which is derived from the cabbage looper moth *Trichoplusia ni* insect [127,128]. In comparison with *SB*, the *PB* transposon was found to be active in a wider variety of species such as insects, yeast and mammals, including humans [129]. The codon optimization of the wild-type insect transposase for expression in **m**ammals (**m**PBase) generated a 20-fold increase in the transposition efficiency [130]. Additionally, the isolation of **hy**peractive transposase variants (**hy**PBase and **bz-hy**PBase) using a screening system in yeast outperformed the transposition efficiencies of the original transposase without compromising its unique features [131,132]. *PB* transposons almost exclusively integrate into TTAA sequences and predominantly display no footprint affecting the genomic integrity at the excision site [133]. This last feature is particularly relevant to generate the induced pluripotent stem cells (iPSCs) in the context of cell-based regenerative medicine, which requires the transient integration of reprogramming genes into the genome of somatic cells, and their subsequent elimination to give rise to any other cell type, aiming at replacing damaged or diseased ones [134,135]. Furthermore, the identification of an excision proficient and integration deficient transposase variant is especially useful to generate the seamless removal of the reprogramming factors from iPS cells without a potential harmful reintegration [136]. The large cargo capacity (superior to 100-kb) offers the possibility to mobilize large genomic fragments, such as those carried by bacterial artificial chromosomes (BACs) in mouse and human embryonic stem cells, which have been used for basic research or to generate transgenic animals [137,138]. Finally, *PB* transposons display a significant bias toward transcriptionally active regions including transcription start sites, CpG islands and DNAseI hypersensitive sites [125,133,139].

***Tc Buster*** **DNA transposons:** *TcBuster*^TM^, the third DNA transposon found in the Gene Therapy toolkit, was identified using bioinformatics tools. Its name is derived from the species from which it was isolated, the red flour beetle ***T****ribolium **c**astaneum* [140]. A high-throughput combinatorial library method was used to generate a highly active transposase protein, TcB-M, outperforming the previous mutants in various mammalian cells, including T and Natural Killer (NK) cells [141,142]. *TcBuster* transposon excision sites are repaired by nonhomologous end-joining [143]. The characterization of *TcBuster* integration sites revealed a strong preference for TA nucleotide sequences, representing over 93% analyzed insertion sequences, and two major secondary sites displaying TG or CA changes at the central integration site [143,144]. *TcBuster* transposons slightly favor transcription units, CpG islands, transcription start sites and preferential cleavage sites for DNase I [143,144].

**Ongoing clinical trials using DNA transposons:** *SB* was the first DNA transposon to enter the clinical stage in 2011. To date, nearly two dozen phase I or I/II clinical trials have been registered using either the *SB* or *PB* transposons to modify cells of the immune system for the expression of chimeric antigen receptors (CARs) (see Section 8), mostly covering the field of adoptive immunotherapy for hematological malignancies, metastatic breast, prostate and lung cancers, as well as metastatic solid tumors. In addition, *SB* transposon-engineered plasmablasts expressing α-I-iduronidase are currently evaluated to treat patients suffering from Hurler syndrome [145,146]. The first phase I clinical trial using the *TcBuster* transposon is currently under development at the stage of patient recruitment (# NCT05312801) for Non-Hodgkin Lymphoma. The therapeutic candidate consists of autologous CAR-T cell therapy using CD4 and CD8 positive human cells that are genetically engineered to target the B cell activating factor receptor (BAFF) to eliminate malignant B cells for the treatment of patients with relapsed or refractory non-Hodgkin lymphoma.

Over the last two decades, no cases of malignant cells derived from either retro, lenti or *SB*-modified T cells have been reported in hundreds of treated patients, thus revealing the safety associated with efficacy records. It is worth mentioning that during a phase I clinical trial of CD19-targeted allogenic CAR-T cells in 10 patients with relapsed or persistent B cell malignancies, T-cell lymphoma originating from the genetically modified cells using the *PB* transposon have been detected in two patients. One of the two died of sepsis and multiorgan failure after showing a transient response to the treatment [147,148]. The analyses of malignant cells did not reveal transposon insertions into typical oncogenes. Rather, the development of malignant cells have been attributed to multiple genetic alterations, such as an altered genomic copy number, and point mutations unrelated to the insertion sites but affecting the expression of oncogenes and tumor-suppressor genes, respectively. From their thorough analyses, the authors suggested that a malignant transformation is probably related to the production methodology such as the electroporation settings (a single high-voltage pulse), high concentration of transposon and transposase elements, or the expansion of the genetically modified cells. No correlation between T-cell lymphoma and the use of the *PB* transposon has been established [147,148].

Thus, DNA transposons represent a safe and attractive tool to generate stable genetically engineered cells. Nevertheless, any transgene integration into the host chromosomes exhibits the risk of insertional mutagenesis via the activation of proto-oncogenes or inactivation of tumor-suppressor genes. The identification of novel transposable elements or transposase variants that favor transgene integration away from transcriptional units and oncogenes or into a defined safe harbor locus with a high efficiency remains to be an important objective to exclude or further reduce genotoxic risks.

In addition to the safety profiles, non-viral approaches offer the potential to lower the costly and limited capacity production of viral vectors (see the following section for production costs of CAR-T cells).

## 8. Non-Viral Strategy for Adoptive Immunotherapy

Chimeric antigen receptors (CARs) are expressed as transgenic proteins on the surface of T or Natural Killer (NK) cells that are engineered to recognize a specific target antigen for adoptive cell therapies. The CAR basic structure consists of a single-chain variable fragment (scFv) of a corresponding antibody which provides the receptor specificity. The scFv region is connected to a transmembrane domain, itself linked to proteic domains that mediate activation and costimulatory signals upon contact of lymphocytes with the targeted antigens.

In comparison to T cells, innate immune NK cells display cytotoxic properties independent from the Major Histocompatibilty Complex (MHC), which favors the development of CAR-NK cells from allogenic sources and storage of cryopreserved ‘off-the shelf’ products. Nonetheless, NK cells display an inefficient in vitro expansion, a short half-life in circulation, as well as a higher sensitivity of apoptosis, in addition to being responders to viral infection generating a reduced viral transduction efficiency [149,150].

At first, CAR-T cells have mostly been engineered using viral vectors such as lenti- or retroviruses. Currently, hundreds of patients have beneficiated from adoptive immunotherapy. The unprecedented outcomes resulted in the approval, by the Federal Drug Administration, of six CAR-T cell products (Kymriah, Yescarta, Tecartus, Breyanzi, Abecma and Carvykti, with a cost comprised between 373 and 475 k$), all targeting the CD19 or B cell Maturation Antigens (BCMA) in the field of haematological malignancies [151]. Nevertheless, viral approaches display several drawbacks. Despite the major improvements accomplished to increase the lenti- and retrovirus safety profiles, persistent genotoxicity concerns still remain, which are added to a lengthy, laborious and costly production process that requires extensive testing prior to clinical applications. This is incompatible with an increasing general demand.

A variety of refinements are being implemented to enhance the specificity, efficiency, persistence and safety of CAR-T and CAR-NK cells. To reduce production costs and provide for safer transgene integration, non-viral approaches are currently being evaluated in preclinical assays and phase I/II clinical trials using electroporation and nucleofection as physical delivery techniques (see Section 2) and antibiotic-free gene vectors most often coupled to DNA transposon systems. The reduced size of small plasmids increases the probability of bringing the transposon ITRs closer together, thereby facilitating the formation of the synaptic complex, also called a paired-end complex, that is required to mediate an efficient transposition [138,152,153,154]. Furthermore, reduced-size plasmids offer the possibility of decreasing the plasmid DNA amount for cell transfection, thereby increasing cell viability by mitigating the TLR4 and TLR9-independent stimulation of a type I interferon response observed after the cell transfection with a transposon plasmid in a dose-dependent manner [155]. The use of transposase-encoded mRNA for cell transfection further reduces the cytotoxicity and limits multiple transposon hopping, mediating the chromosomal rearrangement and mutagenesis.

Nanoplasmids were combined to *PB* and *TcBuster* transposons to manufacture CAR-T and CAR-NK cells, respectively [82,156]. In the latter case, primary, donor-derived NK cells were genetically modified to express a CAR targeting the C-type lectin-like molecule-1 (CLL-1/ C-Type Lectin Domain Family 12 Member A, CLEC12A) against acute myeloid leukemia (AML) cell lines and primary AML blasts. Anti-BCMA CAR-T cells manufactured with a nanoplasmid and *PB* transposon (Section 5 and Table 1 #NCT03288493) show a better efficacy, with an increased overall and complete response rate in patients with RRMM, in addition to an equal safety compared to a standard plasmid [83]. The potency of pFAR4 miniplasmids in combination with the SB transposon system has been assessed by transfecting human CD4^+^ and CD8^+^ lymphocytes to generate CAR-T cells products. A high and stable CD19 CAR gene expression was reached, 14 days post transfection, using a pFAR plasmid carrying the *SB* transposon paired with a SB100X-encoded mRNA. When comparing to the standard pT2-based CD19 CAR transposon, pFAR4 plasmids allowed a higher viability of T-cells and were consequently approximately four-fold superior to generating CD19-CAR T cells that display potent cytotoxic antitumor cells functions in vitro [101]. Although the CAR expression from mRNA is transient, the production of CAR-T cells is significantly reduced to 24 h as compared to the 10–14 days required when viruses or DNA transposons are used for the delivery of the CAR transgene. This innovative cost-effective strategy is currently being evaluated in two phase I clinical trials targeting a CD19 and NKG2DL antigen, using natural killer cells from peripheral blood, for B-Lineage Acute Lymphoblastic Leukemia and a metastatic solid tumor treatment (#NCT00995137 and NCT03415100). When paired with DNA transposons such as *PB*, Doggybone linear vectors require additional random DNA sequences (~200-bp) flanking the *PB* ITR on either side of the transposon cassette to enable an effective transposition and generate CAR-T cells, which might be necessary for the transposase protein to efficiently bind to the linearized DNA transposon [68]. To target pancreatic cancer cells, a NK-92MI cell line, derived from NK cells for a stable and localized IL-2 expression, has been genetically modified to express a CAR directed towards the mesothelin glycoprotein using a minicircle combined with the *SB* transposon as gene vectors. In comparison with the plasmid counterparts, the superior electroporated cell viability, engraftment and secretion of both the interferon-γ and granzyme B mediated a significant increased lysis of pancreatic cancer cell lines [157]. Tipanee et al. [158] validated a nonviral approach to generate the potential universal off-the-shelf allogenic CD19-CAR-T cell combining minicircles, *SB* transposon and CRISPR-Cas9 to inactivate the allogenic donor T cell receptor (TCR) expression. In comparison with first-generation plasmids, the higher transposition rate and lower toxicity mediated by the minicircle delivering a CD19 CAR transposon, coupled with an SB100X-encoded mRNA, generated an approximately 3.6-fold higher yield of CD19-CAR T cells after 14 days of culture [153]. Using a similar setting, a phase I/IIa is currently assessing the potential of autologous SLAMF7 CAR-T cells in multiple myeloma (Section 4, Table 1 #NTC NCT04499339).

Thus, non-viral approaches for manufacturing CAR-T or CAR-NK offer attractive alternatives, with the positive asset of a reduced production cost which has been estimated to be 20 times lower for both SB100X mRNA and a minicircle delivering the CAR transgene versus lenti- or γ-retroviruses, from the preparation step of the research cell bank to the product released, as well as including the stability testing [159]. In addition, the non-viral approaches offer the possibility of drug handling and administration in safety level class I facilities.

## 9. Conclusions and Perspectives

Gene and cell therapies offer ultra-precise genetic treatments and are currently on an ascending trend. Nevertheless, improvements are still required to hold promises at an affordable price. The gene therapy products approved so far by the FDA have prices ranging from several hundred thousand to several millions of dollars per patient for a single treatment. These high costs are justified by the complexity of manufacturing these advanced medicinal products based on viral gene delivery vectors, and on the need for pharmaceutical industries to recoup their research and development costs. In addition, innovative gene therapy might find broader applications in frequent diseases.

In spite of the fact that a single-dose gene therapy treatment shows definite advantages over costly lifetime healthcare (even if the requirement of repeat administrations cannot be excluded to date), such expensive therapies are hardly handled by national health care systems or private health insurances. Thus, even in wealthy countries, patients are either denied gene therapy treatments or experience lengthy delays in receiving them, which consequently generates major societal economic and ethical issues. To make these innovative treatments affordable to those that need them, to expand gene therapy use to low- and middle-income countries, and to be able to apply gene therapy to the high prevalence diseases, a number of challenges must be overcome. Above all, ways have to be found to bring down the presently extremely high costs of these gene therapy products.

A typical example of this cost barrier is that of the anticancer CAR-T cells therapy. Within a decade, major breakthroughs have been made in the field of adoptive immunotherapy using CAR-T cell products to treat patients with hematological malignancies and resulted in the approval of six-FDA-approved products, as already mentioned [151]. Unfortunately, however, these outstanding treatments are only affordable in a limited number of countries or medical institutions possessing CAR-T cell development facilities. Consequently, different strategies are being explored and consist of the development of CAR-T cells in academic centers using non-viral gene vectors for a reduction in the production time in a cost-effective manner.

In less than three decades, several types of linear or circular antibiotic-free gene vectors have been developed using various selection and manufacturing strategies. Minicircles, MIDGE, pORT, Nanoplasmids, and pFAR expression vectors have now entered the clinic, mostly in phase I, I/II and II trials. Notably, one of the first antibiotic-free gene vectors, a FGF1-expressing pCOR, was included in a phase III clinical trial. To date, the therapeutic areas of the conducted clinical trials covered various fields including immunotherapy against chronic viral infection or cancer, prophylactic vaccines, eye disease, peripheral vascular disease and hearing disorders. Notably, none of the tested gene vectors generated adverse events in treated patients, thus meeting the phase I objective. Promising results and positive trends were also reported that need to be further confirmed by including a higher number of patients, and enhanced by improving either the administration route or the expression vector.

With the increased number of antibiotic-free vectors that display favorable therapeutic effects in preclinical tests and phase I/II clinical trials, it is anticipated that non-viral approaches will take a larger role and thereby open new avenues. Nevertheless, a number of challenges still needs to be met. Some areas of improvements consist of optimizing both physical and chemical delivery methods to increase the transfection efficiency, to refine the targeting of a specific tissue or cell type, and decrease cell toxicity, a parameter of key relevance when patient primary cells are available in a limited number. DNA transposon or CRISPR-Cas9-mediated transgene site-specific integration with minimized off-target events also remains an open challenge.

Considering the central role of plasmid DNAs either as drug products, ancillary materials for the production of viral particles, or templates for in vitro mRNA synthesis and linear vectors, the improvement of the production process should focus on a decrease in the production cost and timeline without affecting the quality of produced materials. To improve the expression vector yield and/or quality, several optimization axes could be followed. The first one consists of the further genetic modification of the *E. coli* strains used for their production. The *E. coli* genome contains more than 40 insertion sequences (ISs) [160] that are small mobile elements that can transpose from the bacterial chromosome to the produced plasmid [161]. IS transposition may only be detected at the end of the purification process, which consequently leads to the rejection of the pharmaceutical product and costly financial loss. Thus, eliminating any source affecting the plasmid integrity will probably provide a higher purity of the pharmaceutical product. Regarding the minicircle production, one of the remaining challenges is related to a further increase in the recombination efficiency for a quasi-elimination of the parental plasmid, potentially by assessing novel recombinases identified from genome sequence databases. The production of the synthetic expression vector, such as DoggyBone, holds great promise as it precludes the lengthy need to produce and characterize master cell banks. Nevertheless, improvements are required, mainly for their use in combination with DNA transposons. In this field, basic research will probably focus on the identification of a protelomerase that generates and circularizes the generated monomers in a cost-effective manner. Circular expression vectors could benefit from the identification of a smaller origin of replication to further decrease the plasmid size while maintaining a high plasmid copy number and production yield. The production of high plasmid numbers requires bacterial propagation in fermenters that also deserve the optimization of growth conditions to meet the specificity of all antibiotic-free gene vectors and ensure consistent product quality from batch to batch, as well as reliable preclinical tests and clinical trials. Finally, a thorough assessment of the pros and cons of bacteria-produced plasmids versus synthetic double-stranded DNA produced ex cellulo by enzymatic processes is required. Undoubtedly, the enormous energy invested in the development of the safer, more efficient and cost-effective production of expression vectors can only benefit improvements of gene and cell therapy products.

## Figures and Tables

**Table 1:** List of clinical trials using antibiotic-free gene vectors.

Antibiotic-Free Gene Vector	Trial ID	Trial Title	Health Conditions	Phase (Status)
Minicircle	NCT04499339	A Phase I/IIa Clinical Trial to Assess Feasibility, Safety and Antitumor Activity of Autologous SLAMF7 CAR-T Cells in Multiple Myeloma	Multiple Myeloma	I/IIa (Active)
MIDGE	DRKS00005723 ^a^	Phase I Study with non-viral jet-injection-gene transfer of a TNF-α expressing MIDGE-vector in cutaneous metastases of malignant melanoma	Malignant melanoma of skin	I (Recruiting)
	NCT01265368	A Clinical Study to Assess Safety and Efficacy of a Tumor Vaccine in Patients With Advanced Renal Cell Carcinoma (ASET)	Stage IV Renal Cell Cancer	I/II (Completed)
pORT	NCT01045915	Safety and Efficacy Study of Electrotransfer of Plasmid AMEP to Treat Advance or Metastatic Melanoma	Melanoma	I (Terminated)
	NCT01664273	Gene Electrotransfer to Muscle With Plasmid AMEP in Patients With Disseminated Cancer	Metastatic Malignant Neoplasm	I (Terminated)
Nanoplasmids	NCT01707069	A Safety and Immunogenicity Phase I Study of CryJ2-DNA-Lysosomal Associated Membrane Protein (CryJ2 -DNA-LAMP) Plasmid	Allergic Rhinoconjunctivitis	I (Completed)
	NCT01966224	A Safety and Immunogenicity Phase IB Study of CryJ2-DNA-Lysosomal Associated Membrane Protein (CryJ2 -DNA-LAMP) Plasmid Assessing the Long Term Safety of Previously Treated Subjects	Allergic Rhinoconjunctivitis	I (Completed)
	NCT02146781	A Safety and Immunogenicity Phase IC Study of CryJ2 -DNA-LAMP Plasmid Vaccine for Assessment of Intradermal (ID) Route of Administration Using the Biojector 2000 Device	Allergic Rhinitis	I (Completed)
	NCT02301754	INVAC-1 Anti-Cancer hTERT DNA Immunotherapy	Solid Tumors	I (Completed)
	NCT03265717	DNA Plasmid Encoding a Modified Human Telomerase Reverse Transcriptase (hTERT), Invac-1 in Chronic Lymphocytic Leukemia	Leukemia, Lymphocytic, Chronic, B-Cell	II (Terminated)
	NCT03288493	P-BCMA-101 Tscm CAR-T Cells in the Treatment of Patients With Multiple Myeloma	Multiple Myeloma	I/II (Terminated)
	NCT03308045	Evaluation of EYS606 in Patients With Non-infectious Posterior, Intermediate or Panuveitis	Non-infectious Uveitis	I/II (Completed)
	NCT04207983	A 48 Week Study to Evaluate the Efficacy and Safety of Two EYS606 Treatment Regimens in Subjects With Active Chronic Non-infectious Uveitis (CNIU)	Non-infectious Uveitis	II (Completed)
	NCT04515043	EXPLORATORY STUDY Addendum to INVAC1-CT-101 (NCT02301754)	Solid Tumor, Adult	I (Completed)
	NCT04591184	A Clinical Trial of a Prophylactic Plasmid DNA Vaccine for COVID-19 [Covigenix VAX-001] in Adults	SARS-CoV-2	I/II (Recruiting)
	NCT04752722	LEGEND Study: EG-70 in NMIBC Patients BCG-Unresponsive and High-Risk NMIBC Incompletely Treated With BCG or BCG-Naïve	Non-muscle Invasive Bladder Cancer With Carcinoma in Situ; Superficial Bladder Cancer	I/II (Recruiting)
	ACTRN 12613000831785 ^b^	A Phase I, Proof of Concept, Open Label, Escalating Dose Study to Assess the Safety, Tolerability and Immunogenicity of a Herpes Simplex Virus (HSV) Deoxyribonucleic Acid (DNA) Vaccine in Healthy Volunteers	Genital Herpes	I (Completed)
	ACTRN 12615000094572 ^b^	A Phase I/IIa, randomized, double blind, placebo-controlled, parallel group, pilot study to assess the safety and efficacy of a therapeutic HSV-2 DNA vaccine in HSV-2 positive adults	Genital Herpes	I/IIa (Completed)
pCOR	NCT00566657	Efficacy and Safety of XRP0038/NV1FGF in Critical Limb Ischemia Patients With Skin Lesions	Peripheral Vascular Diseases	III (Completed)
pFAR	ACTRN 12618001556235 ^b^	A phase I/II non-randomized, controlled trial, evaluating the safety and efficacy of neurotrophin gene therapy delivered during cochlear implant surgery	Hearing loss, deafness	I/II (Recruiting)

**List of clinical trials as indexed** in the clinicaltrials.gov database (https://www.clinicaltrials.gov (accessed on 20 December 2023)), the German Clinical Trials Register ^a^ (https://drks.de/search/en (accessed on 20 December 2023)) or the Australian New Zealand Clinical Trials Registry ^b^ (https://www.anzctr.org.au (accessed on 20 December 2023)).

## References

[1] Nurk S., Koren S., Rhie A., Rautiainen M., Bzikadze A.V., Mikheenko A., Vollger M.R., Altemose N., Uralsky L., Gershman A. (2022). The Complete Sequence of a Human Genome. Science.

[2] Aganezov S., Yan S.M., Soto D.C., Kirsche M., Zarate S., Avdeyev P., Taylor D.J., Shafin K., Shumate A., Xiao C. (2022). A Complete Reference Genome Improves Analysis of Human Genetic Variation. Science.

[3] Hickey G., Monlong J., Ebler J., Novak A.M., Eizenga J.M., Gao Y., Abel H.J., Antonacci-Fulton L.L., Asri M., Baid G. (2023). Pangenome Graph Construction from Genome Alignments with Minigraph-Cactus. Nat. Biotechnol..

[4] Liao W.W., Asri M., Ebler J., Doerr D., Haukness M., Hickey G., Lu S., Lucas J.K., Monlong J., Abel H.J. (2023). A Draft Human Pangenome Reference. Nature.

[5] Arabi F., Mansouri V., Ahmadbeigi N. (2022). Gene Therapy Clinical Trials, Where Do We Go? An Overview. Biomed. Pharmacother..

[6] Mendell J.R., Al-Zaidy S., Shell R., Arnold W.D., Rodino-Klapac L.R., Prior T.W., Lowes L., Alfano L., Berry K., Church K. (2017). Single-Dose Gene-Replacement Therapy for Spinal Muscular Atrophy. N. Engl. J. Med..

[7] Farhood H., Serbina N., Huang L. (1995). The Role of Dioleoyl Phosphatidylethanolamine in Cationic Liposome Mediated Gene Transfer. Biochim. Biophys. Acta.

[8] Cheng Q., Wei T., Farbiak L., Johnson L.T., Dilliard S.A., Siegwart D.J. (2020). Selective Organ Targeting (SORT) Nanoparticles for Tissue-Specific mRNA Delivery and CRISPR–Cas Gene Editing. Nat. Nanotechnol..

[9] Dilliard S.A., Cheng Q., Siegwart D.J., Desimone J. (2021). On the Mechanism of Tissue-Specific mRNA Delivery by Selective Organ Targeting Nanoparticles. Proc. Natl. Acad. Sci. USA.

[10] Li B., Manan R.S., Liang S.Q., Gordon A., Jiang A., Varley A., Gao G., Langer R., Xue W., Anderson D. (2023). Combinatorial Design of Nanoparticles for Pulmonary mRNA Delivery and Genome Editing. Nat. Biotechnol..

[11] Bansal R., Singh A.K., Gandhi R.P., Pant A.B., Kumar P., Gupta K.C. (2014). Galactomannan-PEI Based Non-Viral Vectors for Targeted Delivery of Plasmid to Macrophages and Hepatocytes. Eur. J. Pharm. Biopharm..

[12] Satkauskas S., Bureau M.F., Puc M., Mahfoudi A., Scherman D., Miklavcic D., Mir L.M. (2002). Mechanisms of in Vivo DNA Electrotransfer: Respective Contribution of Cell Electropermeabilization and DNA Electrophoresis. Mol. Ther..

[13] Kisakov D.N., Belyakov I.M., Kisakova L.A., Yakovlev V.A., Tigeeva E.V., Karpenko L.I. (2024). The Use of Electroporation to Deliver DNA-Based Vaccines. Expert Rev. Vaccines.

[14] Bachy M., Boudet F., Bureau M., Girerd-Chambaz Y., Wils P., Scherman D., Meric C. (2001). Electric Pulses Increase the Immunogenicity of an Influenza DNA Vaccine Injected Intramuscularly in the Mouse. Vaccine.

[15] Broderick K.E., Humeau L.M. (2017). Enhanced Delivery of DNA or RNA Vaccines by Electroporation. Methods Mol. Biol..

[16] Distler J.H.W., Jüngel A., Kurowska-Stolarska M., Michel B.A., Gay R.E., Gay S., Distler O. (2005). Nucleofection: A New, Highly Efficient Transfection Method for Primary Human Keratinocytes. Exp. Dermatol..

[17] Liu F., Song Y.K., Liu D. (1999). Hydrodynamics-Based Transfection in Animals by Systemic Administration of Plasmid DNA. Gene Ther..

[18] Zhang G., Budker V., Wolff J.A. (1999). High Levels of Foreign Gene Expression in Hepatocytes after Tail Vein Injections of Naked Plasmid DNA. Hum. Gene Ther..

[19] Kamimura K., Kanefuji T., Yokoo T., Abe H., Suda T., Kobayashi Y., Zhang G., Aoyagi Y., Liu D. (2014). Safety Assessment of Liver-Targeted Hydrodynamic Gene Delivery in Dogs. PLoS ONE.

[20] Kamimura K., Kanefuji T., Suda T., Yokoo T., Zhang G., Aoyagi Y., Liu D. (2023). Liver Lobe-Specific Hydrodynamic Gene Delivery to Baboons: A Preclinical Trial for Hemophilia Gene Therapy. Mol. Ther. Nucleic Acids.

[21] Clark I.B., Hanania E.G., Stevens J., Gallina M., Fieck A., Brandes R., Palsson B.O., Koller M.R. (2006). Optoinjection for Efficient Targeted Delivery of a Broad Range of Compounds and Macromolecules into Diverse Cell Types. J. Biomed. Opt..

[22] Bez M., Foiret J., Shapiro G., Pelled G., Ferrara K.W., Gazit D. (2019). Nonviral Ultrasound-Mediated Gene Delivery in Small and Large Animal Models. Nat. Protoc..

[23] Sun R.R., Noble M.L., Sun S.S., Song S., Miao C.H. (2014). Development of Therapeutic Microbubbles for Enhancing Ultrasound-Mediated Gene Delivery. J. Control. Release.

[24] Panté N., Kann M. (2002). Nuclear Pore Complex Is Able to Transport Macromolecules with Diameters of ∼39 Nm. Mol. Biol. Cell.

[25] Jamali T., Jamali Y., Mehrbod M., Mofrad M.R.K. (2011). Nuclear Pore Complex. Biochemistry and Biophysics of Nucleocytoplasmic Transport in Health and Disease. Int. Rev. Cell Mol. Biol..

[26] Ahn H.-H., Carrington C., Hu Y., Liu H.-W., Ng C., Nam H., Park A., Stace C., West W., Mao H.-Q. (2021). Nanoparticle-Mediated Tumor Cell Expression of mIL-12 via Systemic Gene Delivery Treats Syngeneic Models of Murine Lung Cancers. Sci. Rep..

[27] Kreiss P., Cameron B., Rangara R., Mailhe P., Aguerre-Charriol O., Airiau M., Scherman D., Crouzet J., Pitard B. (1999). Plasmid DNA Size Does Not Affect the Physicochemical Properties of Lipoplexes but Modulates Gene Transfer Efficiency. Nucleic Acids Res..

[28] Darquet A.-M., Rangara R., Kreiss P., Schwartz B., Naimi S., Delaère P., Crouzet J., Scherman D. (1999). Minicircle: An Improved DNA Molecule for in Vitro and in Vivo Gene Transfer. Gene Ther..

[29] Stenler S., Wiklander O.P.B., Badal-Tejedor M., Turunen J., Nordin J., Hallengärd D., Wahren B., Andaloussi S.E.L., Rutland M.W., Edvard Smith C.I. (2013). Micro-Minicircle Gene Therapy: Implications of Size on Fermentation, Complexation, Shearing Resistance, and Expression. Mol. Ther. Nucleic Acids.

[30] Catanese D.J., Fogg J.M., Schrock D.E., Gilbert B.E., Zechiedrich L. (2012). Supercoiled Minivector DNA Resists Shear Forces Associated with Gene Therapy Delivery. Gene Ther..

[31] European Medicines Agency Site. https://www.ema.europa.eu/en/documents/scientific-guideline/guideline-quality-non-clinical-and-clinical-aspects-gene-therapy-medicinal-products_en.pdf.

[32] European Medicines Agency Site. https://www.ema.europa.eu/en/documents/scientific-guideline/guideline-non-clinical-studies-required-first-clinical-use-gene-therapy-medicinal-products_en.pdf.

[33] Mairhofer J., Cserjan-Puschmann M., Striedner G., Nöbauer K., Razzazi-Fazeli E., Grabherr R. (2010). Marker-Free Plasmids for Gene Therapeutic Applications—Lack of Antibiotic Resistance Gene Substantially Improves the Manufacturing Process. J. Biotechnol..

[34] Chadeuf G., Ciron C., Moullier P., Salvetti A. (2005). Evidence for Encapsidation of Prokaryotic Sequences during Recombinant Adeno-Associated Virus Production and Their in Vivo Persistence after Vector Delivery. Mol. Ther..

[35] Schnödt M., Schmeer M., Kracher B., Krüsemann C., Espinosa L.E., Grünert A., Fuchsluger T., Rischmüller A., Schleef M., Büning H. (2016). DNA Minicircle Technology Improves Purity of Adeno-Associated Viral Vector Preparations. Mol. Ther. Nucleic Acids.

[36] Mccarty D.M., Pereira D.J., Zolotukhin I., Zhou X., Ryan J.H., Muzyczka N., Mccarty D.M., Ryan J.H., Zolutukhin S., Zhou X. (1994). Identification of Linear DNA Sequences That Specifically Bind the Adeno-Associated Virus Rep Protein. J. Virol..

[37] Shafaati M., Saidijam M., Soleimani M., Hazrati F., Mirzaei R., Amirheidari B., Tanzadehpanah H., Karampoor S., Kazemi S., Yavari B. (2021). A Brief Review on DNA Vaccines in the Era of COVID-19. Future Virol..

[38] Darquet A.-M., Cameron B., Wils P., Scherman D., Crouzet J. (1997). A New DNA Vehicle for Nonviral Gene Delivery: Supercoiled Minicircle. Gene Ther..

[39] Bigger B.W., Tolmachov O., Collombet J.M., Fragkos M., Palaszewski I., Coutelle C. (2001). An AraC-Controlled Bacterial Cre Expression System to Produce DNA Minicircle Vectors for Nuclear and Mitochondrial Gene Therapy. J. Biol. Chem..

[40] Nehlsen K., Broll S., Bode J. (2006). Replicating Minicircles: Generation of Nonviral Episomes for the Efficient Modification of Dividing Cells. Gene Ther. Mol. Biol..

[41] Chen Z.Y., He C.Y., Ehrhardt A., Kay M.A. (2003). Minicircle DNA Vectors Devoid of Bacterial DNA Result in Persistent and High-Level Transgene Expression in Vivo. Mol. Ther..

[42] Kay M.A., He C.Y., Chen Z.Y. (2010). A Robust System for Production of Minicircle DNA Vectors. Nat. Biotechnol..

[43] Alves C.P.A., Prazeres D.M.F., Monteiro G.A. (2021). Recombination Efficiency Measurement by Real-Time PCR: A Strategy to Evaluate ParA-Mediated Minicircle Production. Anal. Biochem..

[44] Mayrhofer P., Blaesen M., Schleef M., Jechlinger W. (2008). Minicircle-DNA Production by Site Specific Recombination and Protein—DNA Interaction Chromatography. J. Gene Med..

[45] Almeida A.M., António Queiroz J., Sousa F., Sousa Â. (2020). Minicircle DNA: The Future for DNA-Based Vectors?. Trends Biotechnol..

[46] Wils P., Escriou V., Warnery A., Lacroix F., Lagneaux D., Ollivier M., Crouzet J., Mayaux J.-F., Scherman D. (1997). Efficient Purification of Plasmid DNA for Gene Transfer Using Triple-Helix Affinity Chromatography. Gene Ther..

[47] Hou X.H., Guo X.Y., Chen Y., He C.Y., Chen Z.Y. (2015). Increasing the Minicircle DNA Purity Using an Enhanced Triplex DNA Technology to Eliminate DNA Contaminants. Mol. Ther. Methods Clin. Dev..

[48] Alves C.P.A., Šimčíková M., Brito L., Monteiro G.A., Prazeres D.M.F. (2016). Development of a Nicking Endonuclease-Assisted Method for the Purification of Minicircles. J. Chromatogr. A.

[49] Alves C.P.A., Šimčíková M., Brito L., Monteiro G.A., Prazeres D.M.F. (2018). Production and Purification of Supercoiled Minicircles by a Combination of in Vitro Endonuclease Nicking and Hydrophobic Interaction Chromatography. Hum. Gene Ther. Methods.

[50] Madeira C., Rodrigues C.A.V., Reis M.S.C., Ferreira F.F.C.G., Correia R.E.S.M., Diogo M.M., Cabral J.M.S. (2013). Nonviral Gene Delivery to Neural Stem Cells with Minicircles by Microporation. Biomacromolecules.

[51] Wang X., Alshehri F., Manzanares D., Li Y., He Z., Qiu B., Zeng M., A. S., Lara-Sáez I., Wang W. (2021). Development of Minicircle Vectors Encoding Col7a1 Gene with Human Promoters for Non-Viral Gene Therapy for Recessive Dystrophic Epidermolysis Bullosa. Int. J. Mol. Sci..

[52] Florian M., Wang J.P., Deng Y., Souza-Moreira L., Stewart D.J., Mei S.H.J. (2021). Gene Engineered Mesenchymal Stem Cells: Greater Transgene Expression and Efficacy with Minicircle vs. Plasmid DNA Vectors in a Mouse Model of Acute Lung Injury. Stem Cell Res. Ther..

[53] Serra J., Alves C.P.A., Brito L., Monteiro G.A., Cabral J.M.S., Prazeres D.M.F., Da Silva C.L. (2019). Engineering of Human Mesenchymal Stem/Stromal Cells with Vascular Endothelial Growth Factor-Encoding Minicircles for Angiogenic Ex Vivo Gene Therapy. Hum. Gene Ther..

[54] Narsinh K.H., Jia F., Robbins R.C., Kay M.A., Longaker M.T., Wu J.C. (2011). Generation of Adult Human Induced Pluripotent Stem Cells Using Nonviral Minicircle DNA Vectors. Nat. Protoc..

[55] Prommersberger S., Reiser M., Beckmann J., Danhof S., Amberger M., Quade-Lyssy P., Einsele H., Hudecek M., Bonig H., Ivics Z. (2021). CARAMBA: A First-in-Human Clinical Trial with SLAMF7 CAR-T Cells Prepared by Virus-Free Sleeping Beauty Gene Transfer to Treat Multiple Myeloma. Gene Ther..

[56] Schakowski F., Gorschlüter M., Buttgereit P., Märten A., Lilienfeld-Toal M.V., Junghans C., Schroff M., König-Merediz S.A., Ziske C., Strehl J. (2007). Minimal Size MIDGE Vectors Improve Transgene Expression In Vivo. In Vivo.

[57] Leutenegger C.M., Boretti F.S., Mislin C.N., Flynn J.N., Schroff M., Habel A., Junghans C., Koenig-Merediz S.A., Sigrist B., Aubert A. (2000). Immunization of Cats against Feline Immunodeficiency Virus (FIV) Infection by Using Minimalistic Immunogenic Defined Gene Expression Vector Vaccines Expressing FIV Gp140 Alone or with Feline Interleukin-12 (IL-12), IL-16, or a CpG Motif. J. Virol..

[58] Boretti F.S., Leutenegger C.M., Mislin C., Hofmann-Lehmann R., Ko S., Schroff M., Junghans C., Fehr D., Huettner S.W., Habel A.Â. (2000). Protection against FIV Challenge Infection by Genetic Vaccination Using Minimalistic DNA Constructs for FIV Env Gene and Feline IL-12 Expression. AIDS.

[59] López-Fuertes L., Pérez-Jiménez E., Vila-Coro A.J., Sack F., Moreno S., Konig S.A., Junghans C., Wittig B., Timón M., Esteban M. (2002). DNA Vaccination with Linear Minimalistic (MIDGE) Vectors Confers Protection against Leishmania Major Infection in Mice. Vaccine.

[60] Endmann A., Baden M., Weisermann E., Kapp K., Schroff M., Kleuss C., Wittig B., Juhls C. (2010). Immune Response Induced by a Linear DNA Vector: Influence of Dose, Formulation and Route of Injection. Vaccine.

[61] Schirmbeck R., König-Merediz S.A., Riedl P., Kwissa M., Sack F., Schroff M., Junghans C., Reimann J., Wittig B. (2001). Priming of Immune Responses to Hepatitis B Surface Antigen with Minimal DNA Expression Constructs Modified with a Nuclear Localization Signal Peptide. J. Mol. Med..

[62] Zheng C., Juhls C., Oswald D., Sack F., Westfehling I., Wittig B., Babiuk L.A., van Drunen Littel-van den Hurk S. (2006). Effect of Different Nuclear Localization Sequences on the Immune Responses Induced by a MIDGE Vector Encoding Bovine Herpesvirus-1 Glycoprotein D. Vaccine.

[63] Volz B., Schmidt M., Heinrich K., Kapp K., Schroff M., Wittig B. (2016). Design and Characterization of the Tumor Vaccine MGN1601, Allogeneic Fourfold Gene-Modified Vaccine Cells Combined with a TLR-9 Agonist. Mol. Ther. Oncolytics.

[64] Nafissi N., Alqawlaq S., Lee E.A., Foldvari M., Spagnuolo P.A., Slavcev R.A. (2014). DNA Ministrings: Highly Safe and Effective Gene Delivery Vectors. Mol. Ther. Nucleic Acids.

[65] Wong S., Lam P., Nafissi N., Denniss S., Slavcev R. (2016). Production of Double-Stranded DNA Ministrings. J. Vis. Exp..

[66] Barreira M., Kerridge C., Jorda S., Olofsson D., Neumann A., Horton H., Smith-Moore S. (2023). Enzymatically Amplified Linear DbDNA^TM^ as a Rapid and Scalable Solution to Industrial Lentiviral Vector Manufacturing. Gene Ther..

[67] Mucker E.M., Brocato R.L., Principe L.M., Kim R.K., Zeng X., Smith J.M., Kwilas S.A., Kim S., Horton H., Caproni L. (2022). SARS-CoV-2 Doggybone DNA Vaccine Produces Cross-Variant Neutralizing Antibodies and Is Protective in a COVID-19 Animal Model. Vaccines.

[68] Bishop D.C., Caproni L., Gowrishankar K., Legiewicz M., Karbowniczek K., Tite J., Gottlieb D.J., Micklethwaite K.P. (2020). CAR T Cell Generation by PiggyBac Transposition from Linear Doggybone DNA Vectors Requires Transposon DNA-Flanking Regions. Mol. Ther. Methods Clin. Dev..

[69] Grabherr R., Bayer K. (2002). Impact of Targeted Vector Design on Col E1 Plasmid Replication. Trends Biotechnol..

[70] Filutowicz M., McEachern M.J., Mukhopadhyay P., Greener A., Yang S.L., Helinski D.R. (1987). DNA and Protein Interactions in The Regulation of Plasmid Replication. J. Cell Sci. Suppl..

[71] Soubrier F., Cameron B., Manse B., Somarriba S., Dubertret C., Jaslin G., Jung G., Le Caer C., Dang D., Mouvault J.M. (1999). pCOR: A New Design of Plasmid Vectors for Nonviral Gene Therapy. Gene Ther..

[72] Soubrier F., Laborderie B., Cameron B. (2005). Improvement of pCOR Plasmid Copy Number for Pharmaceutical Applications. Appl. Microbiol. Biotechnol..

[73] Cranenburgh R.M., Hanak J.A.J., Williams S.G., Sherratt D.J. (2001). *Escherichia coli* Strains That Allow Antibiotic-Free Plasmid Selection and Maintenance by Repressor Titration. Nucleic Acids Res..

[74] Cranenburgh R.M., Lewis K.S., Hanak J.A.J. (2004). Effect of Plasmid Copy Number and Lac Operator Sequence on Antibiotic-Free Plasmid Selection by Operator-Repressor Titration in Escherichia Coli. J. Mol. Microbiol. Biotechnol..

[75] Ramos I., Alonso A., Peris A., Marcen J.M., Abengozar M.A., Alcolea P.J., Castillo J.A., Larraga V. (2009). Antibiotic Resistance Free Plasmid DNA Expressing LACK Protein Leads towards a Protective Th1 Response against Leishmania Infantum Infection. Vaccine.

[76] Kamensek U., Rencelj A., Jesenko T., Remic T., Sersa G., Cemazar M. (2022). Maintenance and Gene Electrotransfer Efficiency of Antibiotic Resistance Gene-Free Plasmids Encoding Mouse, Canine and Human Interleukin-12 Orthologues. Heliyon.

[77] Spanggaard I., Snoj M., Cavalcanti A., Bouquet C., Sersa G., Robert C., Cemazar M., Dam E., Vasseur B., Attali P. (2013). Gene Electrotransfer of Plasmid Antiangiogenic Metargidin Peptide (AMEP) in Disseminated Melanoma: Safety and Efficacy Results of a Phase i First-in-Man Study. Hum. Gene Ther. Clin. Dev..

[78] Spanggaard I., Dahlstroem K., Laessoee L., Hansen R.H., Johannesen H.H., Hendel H.W., Bouquet C., Attali P., Gehl J. (2017). Gene Therapy for Patients with Advanced Solid Tumors: A Phase I Study Using Gene Electrotransfer to Muscle with the Integrin Inhibitor Plasmid AMEP. Acta Oncol..

[79] Mairhofer J., Pfaffenzeller I., Merz D., Grabherr R. (2008). A Novel Antibiotic Free Plasmid Selection System: Advances in Safe and Efficient DNA Therapy. Biotechnol. J..

[80] Carnes A.E., Luke J.M., Vincent J.M., Anderson S., Schukar A., Hodgson C.P., Williams J.A. (2010). Critical Design Criteria for Minimal Antibiotic-Free Plasmid Vectors Necessary to Combine Robust RNA Pol II and Pol III-Mediated Eukaryotic Expression with High Bacterial Production Yields. J. Gene Med..

[81] Luke J., Carnes A.E., Hodgson C.P., Williams J.A. (2009). Improved Antibiotic-Free DNA Vaccine Vectors Utilizing a Novel RNA Based Plasmid Selection System. Vaccine.

[82] Costello C., Derman B.A., Kocoglu M.H., Deol A., Ali A.A., Gregory T., Dholaria B., Berdeja J.G., Cohen A.D., Patel K.K. (2021). Clinical Trials of BCMA-Targeted CAR-T Cells Utilizing a Novel Non-Viral Transposon System. Blood.

[83] Ostertag E. Manufacturing Matters in CAR-T: Small Changes Can Have a Big Impact. Poseida Therapeutics Site. https://poseida.com/wp-content/uploads/2021/01/Manufacturing-Matters-in-CAR-T.pdf.

[84] Su Y., Romeu-Bonilla E., Anagnostou A., Fitz-Patrick D., Hearl W., Heiland T. (2017). Safety and Long-Term Immunological Effects of CryJ2-LAMP Plasmid Vaccine in Japanese Red Cedar Atopic Subjects: A Phase I Study. Hum. Vaccines Immunother..

[85] Teixeira L., Medioni J., Garibal J., Adotevi O., Doucet L., Durey M.A.D., Ghrieb Z., Kiladjian J.J., Brizard M., Laheurte C. (2020). A First-in-Human Phase I Study of INVAC-1, an Optimized Human Telomerase DNA Vaccine in Patients with Advanced Solid Tumors. Clin. Cancer Res..

[86] Hoogewoud F., Buggage R., Behar-Cohen F. (2019). EYS606 for the Treatment of Non-Infectious Uveitis. Acta Ophthalmol..

[87] Buggage R., Behar-Cohen F. (2020). EYS606 for the Treatment of Chronic Non-Infectious Uveitis (NIU): Results from Part 1 of a First-in-Human (EYS606-CT1) Study. Investig. Ophthalmol. Vis. Sci..

[88] Luke J.M., Simon G.G., Söderholm J., Errett J.S., August J.T., Gale M., Hodgson C.P., Williams J.A. (2011). Coexpressed RIG-I Agonist Enhances Humoral Immune Response to Influenza Virus DNA Vaccine. J. Virol..

[89] Steinberg G.D., Kalota S.J., Lotan Y., Warner L., Dauphinee S., Mazanet R. (2023). Clinical Results of a Phase 1 Study of Intravesical EG-70 in Patients with BCG-Unresponsive NMIBC. J. Clin. Oncol..

[90] Chandra J., Woo W.P., Dutton J.L., Xu Y., Li B., Kinrade S., Druce J., Finlayson N., Griffin P., Laing K.J. (2019). Immune Responses to a HSV-2 Polynucleotide Immunotherapy COR-1 in HSV-2 Positive Subjects: A Randomized Double Blinded Phase I/IIa Trial. PLoS ONE.

[91] Marie C., Vandermeulen G., Quiviger M., Richard M., Préat V., Scherman D. (2010). PFARs, Plasmids Free of Antibiotic Resistance Markers, Display High-Level Transgene Expression in Muscle, Skin and Tumour Cells. J. Gene Med..

[92] Belch J., Hiatt W.R., Baumgartner I., Driver V., Nikol S., Norgren L., Van Belle E. (2011). Effect of Fibroblast Growth Factor NV1FGF on Amputation and Death: A Randomised Placebo-Controlled Trial of Gene Therapy in Critical Limb Ischaemia. Lancet.

[93] Bakker N.A.M., de Boer R., Marie C., Scherman D., Haanen J.B.A.G., Beijnen J.H., Nuijen B., van den Berg J.H. (2019). Small-Scale GMP Production of Plasmid DNA Using a Simplified and Fully Disposable Production Method. J. Biotechnol..

[94] Hernandez M., Recalde S., Garcia-Garcia L., Bezunartea J., Miskey C., Johnen S., Diarra S., Sebe A., Rodriguez-Madoz J.R., Pouillot S. (2019). Preclinical Evaluation of a Cell-Based Gene Therapy Using the Sleeping Beauty Transposon System in Choroidal Neovascularization. Mol. Ther. Methods Clin. Dev..

[95] Garcia-Garcia L., Recalde S., Hernandez M., Bezunartea J., Rodriguez-Madoz J.R., Johnen S., Diarra S., Marie C., Izsvák Z., Ivics Z. (2017). Long-Term PEDF Release in Rat Iris and Retinal Epithelial Cells after Sleeping Beauty Transposon-Mediated Gene Delivery. Mol. Ther. Nucleic Acids.

[96] Pastor M., Johnen S., Harmening N., Quiviger M., Pailloux J., Kropp M., Walter P., Ivics Z., Izsvák Z., Thumann G. (2018). The Antibiotic-Free pFAR4 Vector Paired with the Sleeping Beauty Transposon System Mediates Efficient Transgene Delivery in Human Cells. Mol. Ther. Nucleic Acids.

[97] Johnen S., Harmening N., Marie C., Scherman D., Izsvák Z., Ivics Z., Walter P., Thumann G. (2021). Electroporation-Based Genetic Modification of Primary Human Pigment Epithelial Cells Using the Sleeping Beauty Transposon System. J. Vis. Exp..

[98] Thumann G., Harmening N., Prat-Souteyrand C., Marie C., Pastor M., Sebe A., Miskey C., Hurst L.D., Diarra S., Kropp M. (2017). Engineering of PEDF-Expressing Primary Pigment Epithelial Cells by the SB Transposon System Delivered by pFAR4 Plasmids. Mol. Ther. Nucleic Acids.

[99] Quiviger M., Arfi A., Mansard D., Delacotte L., Pastor M., Scherman D., Marie C. (2014). High and Prolonged Sulfamidase Secretion by the Liver of MPS-IIIA Mice Following Hydrodynamic Tail Vein Delivery of Antibiotic-Free pFAR4 Plasmid Vector. Gene Ther..

[100] Pastor M., Quiviger M., Pailloux J., Scherman D., Marie C. (2020). Reduced Heterochromatin Formation on the pFAR4 Miniplasmid Allows Sustained Transgene Expression in the Mouse Liver. Mol. Ther. Nucleic Acids.

[101] Gogishvili T., Monjezi R., Marie C., Machwirth M., Einsele H., Ivics Z., Scherman D., Hudecek M. Enhanced engineering of chimeric antigen receptor (CAR)-modified T Cells using non-viral Sleeping Beauty transposition from pFAR vectors. Proceedings of the European Society of Gene and Cell Therapy.

[102] Zabaleta N., Hommel M., Salas D., Gonzalez-Aseguinolaza G. (2019). Genetic-Based Approaches to Inherited Metabolic Liver Diseases. Hum. Gene Ther..

[103] Riu E., Chen Z.-Y., Xu H., He C.-Y., Kay M.A. (2007). Histone Modifications Are Associated with the Persistence or Silencing of Vector-Mediated Transgene Expression in Vivo. Mol. Ther..

[104] Aliño S.F., Crespo A., Dasí F. (2003). Long-Term Therapeutic Levels of Human Alpha-1 Antitrypsin in Plasma after Hydrodynamic Injection of Nonviral DNA. Gene Ther..

[105] Miao C.H., Ohashi K., Patijn G.A., Meuse L., Ye X., Thompson A.R., Kay M.A. (2000). Inclusion of the Hepatic Locus Control Region, an Intron, and Untranslated Region Increases and Stabilizes Hepatic Factor IX Gene Expression in Vivo but Not in Vitro. Mol. Ther..

[106] Chen Z.Y., He C.-Y., Meuse L., Kay M.A. (2004). Silencing of Episomal Transgene Expression by Plasmid Bacterial DNA Elements in Vivo. Gene Ther..

[107] Lu J., Zhang F., Xu S., Fire A.Z., Kay M.A. (2012). The Extragenic Spacer Length between the 5′ and 3′ Ends of the Transgene Expression Cassette Affects Transgene Silencing from Plasmid-Based Vectors. Mol. Ther..

[108] Lu J., Zhang F., Fire A.Z., Kay M.A. (2017). Sequence-Modified Antibiotic Resistance Genes Provide Sustained Plasmid-Mediated Transgene Expression in Mammals. Mol. Ther..

[109] Gracey Maniar L.E., Maniar J.M., Chen Z.Y., Lu J., Fire A.Z., Kay M.A. (2013). Minicircle DNA Vectors Achieve Sustained Expression Reflected by Active Chromatin and Transcriptional Level. Mol Ther..

[110] Segal E., Widom J. (2009). Poly(DA:DT) Tracts: Major Determinants of Nucleosome Organization. Curr. Opin. Struct. Biol..

[111] Mendenhall E.M., Koche R.P., Truong T., Zhou V.W., Issac B., Chi A.S., Ku M., Bernstein B.E. (2010). GC-Rich Sequence Elements Recruit PRC2 in Mammalian ES Cells. PLoS Genet..

[112] Kropp M., Harmening N., Bascuas T., Johnen S., De Clerck E., Fernández V., Ronchetti M., Cadossi R., Zanini C., Scherman D. (2022). GMP-Grade Manufacturing and Quality Control of a Non-Virally Engineered Advanced Therapy Medicinal Product for Personalized Treatment of Age-Related Macular Degeneration. Biomedicines.

[113] Pinyon J.L., von Jonquieres G., Crawford E.N., Duxbury M., Al Abed A., Lovell N.H., Klugmann M., Wise A.K., Fallon J.B., Shepherd R.K. (2019). Neurotrophin Gene Augmentation by Electrotransfer to Improve Cochlear Implant Hearing Outcomes. Hear. Res..

[114] Pinyon J.L., Tadros S.F., Froud K.E., Wong A.C.Y., Tompson I.T., Crawford E.N., Ko M., Morris R., Klugmann M., Housley G.D. (2014). Close-Field Electroporation Gene Delivery Using the Cochlear Implant Electrode Array Enhances the Bionic Ear. Sci. Transl. Med..

[115] Wang Z., Troilo P.J., Wang X., Griffiths T.G., Pacchione S.J., Barnum A.B., Harper L.B., Pauley C.J., Niu Z., Denisova L. (2004). Detection of Integration of Plasmid DNA into Host Genomic DNA Following Intramuscular Injection and Electroporation. Gene Ther..

[116] Ledwith B.J., Manam S., Troilo P.J., Barnum A.B., Pauley C.J., Griffiths T.G., Harper L.B., Beare C.M., Bagdon W.J., Nichols W.W. (2000). Plasmid DNA Vaccines: Investigation of Integration into Host Cellular DNA Following Intramuscular Injection in Mice. Intervirology.

[117] Mcclintock B. (1950). The Origin and Behavior of Mutable Loci in Maize. Proc. Natl. Acad. Sci. USA.

[118] Lander S., Linton L.M., Birren B., Nusbaum C., Zody M.C., Baldwin J., Devon K., Dewar K., Doyle M., FitzHugh W. (2001). Initial Sequencing and Analysis of the Human Genome. Nature.

[119] Ivics Z., Hackett P.B., Plasterk R.H., Izsvák Z. (1997). Molecular Reconstruction of Sleeping Beauty, a Tc1-like Transposon from Fish, and Its Transposition in Human Cells. Cell.

[120] Mátés L., Chuah M.K.L., Belay E., Jerchow B., Manoj N., Acosta-Sanchez A., Grzela D.P., Schmitt A., Becker K., Matrai J. (2009). Molecular Evolution of a Novel Hyperactive Sleeping Beauty Transposase Enables Robust Stable Gene Transfer in Vertebrates. Nat. Genet..

[121] Izsvák Z., Stüwe E.E., Fiedler D., Katzer A., Jeggo P.A., Ivics Z.N. (2004). Healing the Wounds Inflicted by Sleeping Beauty Transposition by Double-Strand Break Repair in Mammalian Somatic. Mol. Cell.

[122] Vigdal T.J., Kaufman C.D., Izsvák Z., Voytas D.F., Ivics Z. (2002). Common Physical Properties of DNA Affecting Target Site Selection of Sleeping Beauty and Other Tc1/Mariner Transposable Elements. J. Mol. Biol..

[123] Grabundzija I., Irgang M., Mátés L., Belay E., Matrai J., Gogol-Döring A., Kawakami K., Chen W., Ruiz P., Chuah M.K.L. (2010). Comparative Analysis of Transposable Element Vector Systems in Human Cells. Mol. Ther..

[124] Zayed H., Izsvák Z., Walisco O., Ivics Z. (2004). Development of Hyperactive Sleeping Beauty Transposon Vectors by Mutational Analysis. Mol. Ther..

[125] Gogol-Doring A., Ammar I., Gupta S., Bunse M., Miskey C., Chen W., Uckert W., Schulz T.F., Izsvak Z., Ivics Z. (2016). Genome-Wide Profiling Reveals Remarkable Parallels between Insertion Site Selection Properties of the MLV Retrovirus and the PiggyBac Transposon in Primary Human CD4^+^ T Cells. Mol. Ther..

[126] Miskey C., Kesselring L., Querques I., Abrusán G., Barabas O., Ivics Z. (2022). Engineered Sleeping Beauty Transposase Redirects Transposon Integration Away from Genes. Nucleic Acids Res..

[127] Cary L.C., Goebel M., Corsaro B.G., Wang H.-G., Rosen E., Fraser M.J. (1989). Transposon Mutagenesis of Baculoviruses: Analysis of Trichoplusia Ni Transposon IFP2 Insertions within the FP-Locus of Nuclear Polyhedrosis Viruses. Virology.

[128] Elick T.A., Bauser C.A., Fraser M.J. (1996). Excision of the PiggyBac Transposable Element in Vitro Is a Precise Event That Is Enhanced by the Expression of Its Encoded Transposase. Genetica.

[129] Ding S., Wu X., Li G., Han M., Zhuang Y., Xu T. (2005). Efficient Transposition of the PiggyBac (PB) Transposon in Mammalian Cells and Mice. Cell.

[130] Cadiñanos J., Bradley A. (2007). Generation of an Inducible and Optimized PiggyBac Transposon Systemy. Nucleic Acids Res..

[131] Wen W., Song S., Han Y., Chen H., Liu X., Qian Q. (2020). An Efficient Screening System in Yeast to Select a Hyperactive Piggybac Transposase for Mammalian Applications. Int. J. Mol. Sci..

[132] Yusa K., Zhou L., Li M.A., Bradley A., Craig N.L. (2011). A Hyperactive PiggyBac Transposase for Mammalian Applications. Proc. Natl. Acad. Sci. USA.

[133] Galvan D.L., Nakazawa Y., Kaja A., Kettlun C., Cooper L.J.N., Rooney C.M., Wilson M.H. (2009). Genome-Wide Mapping of Piggybac Transposon Integrations in Primary Human T Cells. J. Immunother..

[134] Takahashi K., Yamanaka S. (2006). Induction of Pluripotent Stem Cells from Mouse Embryonic and Adult Fibroblast Cultures by Defined Factors. Cell.

[135] Yusa K., Rad R., Takeda J., Bradley A. (2009). Generation of Transgene-Free Induced Pluripotent Mouse Stem Cells by the PiggyBac Transposon. Nat. Methods.

[136] Li X., Burnight E.R., Cooney A.L., Malani N., Brady T., Sander J.D., Staber J., Wheelan S.J., Joung J.K., McCray P.B. (2013). PiggyBac Transposase Tools for Genome Engineering. Proc. Natl. Acad. Sci. USA.

[137] Li R., Zhuang Y., Han M., Xu T., Wu X. (2013). PiggyBac as a High-Capacity Transgenesis and Gene-Therapy Vector in Human Cells and Mice. Dis. Models Mech..

[138] Rostovskaya M., Fu J., Obst M., Baer I., Weidlich S., Wang H., Smith A.J.H., Anastassiadis K., Francis Stewart A. (2012). Transposon-Mediated BAC Transgenesis in Human ES Cells. Nucleic Acids Res..

[139] Huang X., Guo H., Tammana S., Jung Y.C., Mellgren E., Bassi P., Cao Q., Tu Z.J., Kim Y.C., Ekker S.C. (2010). Gene Transfer Efficiency and Genome-Wide Integration Profiling of Sleeping Beauty, Tol2, and PiggyBac Transposons in Human Primary T Cells. Mol. Ther..

[140] Richards S., Gibbs R.A., Weinstock G.M., Brown S., Denell R., Beeman R.W., Bucher G., Friedrich M., Grimmelikhuijzen C.J.P., Klingler M. (2008). The Genome of the Model Beetle and Pest Tribolium Castaneum. Nature.

[141] Patrinostro X., Hermanson D., Silaika S. (2022). Accelerating Cell Therapy Discovery and Development with Non-Viral Gene Engineering. Cell Gene Ther. Insights.

[142] Pomeroy E.J., Lahr W.S., Chang J.W., Krueger J., Wick B.J., Slipek N.J., Skeate J.G., Webber B.R., Moriarity B.S. (2021). Non-Viral Engineering of CAR-NK and CAR-T Cells Using the Tc Buster Transposon System^TM^. BioRxiv.

[143] Woodard L.E., Li X., Malani N., Kaja A., Hice R.H., Atkinson P.W., Bushman F.D., Craig N.L., Wilson M.H. (2012). Comparative Analysis of the Recently Discovered hAT Transposon TcBuster in Human Cells. PLoS ONE.

[144] Li X., Ewis H., Hice R.H., Malani N., Parker N., Zhou L., Feschotte C., Bushman F.D., Atkinson P.W., Craig N.L. (2013). A Resurrected Mammalian hAT Transposable Element and a Closely Related Insect Element Are Highly Active in Human Cell Culture. Proc. Natl. Acad. Sci. USA.

[145] Amberger M., Ivics Z. (2020). Latest Advances for the Sleeping Beauty Transposon System: 23 Years of Insomnia but Prettier than Ever: Refinement and Recent Innovations of the Sleeping Beauty Transposon System Enabling Novel, Nonviral Genetic Engineering Applications. BioEssays.

[146] Yagyu S., Nakazawa Y. (2023). PiggyBac-Transposon-Mediated CAR-T Cells for the Treatment of Hematological and Solid Malignancies. Int. J. Clin. Oncol..

[147] Micklethwaite K.P., Gowrishankar K., Gloss B.S., Li Z., Street J.A., Moezzi L., Mach M.A., Sutrave G., Clancy L.E., Bishop D.C. (2021). Investigation of product-derived lymphoma following infusion of piggyBac-modified CD19 chimeric antigen receptor T cells. Blood.

[148] Bishop D.C., Clancy L.E., Renee Simms R., Burgess J., Mathew G., Moezzi L., Street J.A., Sutrave G., Atkins E., McGuire H.M. (2021). Development of CAR T-cell lymphoma in 2 of 10 patients effectively treated with piggyBac-modified CD19 CAR T cells. Blood.

[149] Dagher O.K., Posey A.D. (2023). Forks in the Road for CAR T and CAR NK Cell Cancer Therapies. Nat. Immunol..

[150] Balke-Want H., Keerthi V., Cadinanos-Garai A., Fowler C., Gkitsas N., Brown A.K., Tunuguntla R., Abou-el-Enein M., Feldman S.A. (2023). Non-Viral Chimeric Antigen Receptor (CAR) T Cells Going Viral. Immuno-Oncol. Technol..

[151] Witkowsky L., Norstad M., Glynn A.R., Kliegman M. (2023). Towards Affordable CRISPR Genomic Therapies: A Task Force Convened by the Innovative Genomics Institute. Gene Ther..

[152] Izsvák Z., Ivics Z., Plasterk R.H. (2000). Sleeping Beauty, a Wide Host-Range Transposon Vector for Genetic Trensformation in Vertebrates. J. Mol. Biol..

[153] Monjezi R., Miskey C., Gogishvili T., Schleef M., Schmeer M., Einsele H., Ivics Z., Hudecek M. (2017). Enhanced CAR T-Cell Engineering Using Non-Viral Sleeping Beauty Transposition from Minicircle Vectors. Leukemia.

[154] Jin Y., Chen Y., Zhao S., Guan K.L., Zhuang Y., Zhou W., Wu X., Xu T. (2017). DNA-PK Facilitates PiggyBac Transposition by Promoting Paired-End Complex Formation. Proc. Natl. Acad. Sci. USA.

[155] Clauss J., Obenaus M., Miskey C., Ivics Z., Izsvák Z., Uckert W., Bunse M. (2018). Efficient Non-Viral T-Cell Engineering by Sleeping Beauty Minicircles Diminishing DNA Toxicity and miRNAs Silencing the Endogenous T-Cell Receptors. Hum. Gene Ther..

[156] Gurney M., O’Reilly E., Corcoran S., Brophy S., Krawczyk J., Otto N.M., Hermanson D.L., Childs R.W., Szegezdi E., O’Dwyer M.E. (2022). Concurrent Transposon Engineering and CRISPR/Cas9 Genome Editing of Primary CLL-1 Chimeric Antigen Receptor–Natural Killer Cells. Cytotherapy.

[157] Batchu R.B., Gruzdyn O.V., Tavva P.S., Kolli B.K., Dachepalli R., Weaver D.W., Gruber S.A. (2019). Engraftment of Mesothelin Chimeric Antigen Receptor Using a Hybrid Sleeping Beauty/Minicircle Vector into NK-92MI Cells for Treatment of Pancreatic Cancer. Surgery.

[158] Tipanee J., Samara-Kuko E., Gevaert T., Chuah M.K., VandenDriessche T. (2022). Universal Allogeneic CAR T Cells Engineered with Sleeping Beauty Transposons and CRISPR-CAS9 for Cancer Immunotherapy. Mol. Ther..

[159] Hudecek M., Ivics Z. (2018). Non-Viral Therapeutic Cell Engineering with the Sleeping Beauty Transposon System. Curr. Opin. Genet. Dev..

[160] Blattner F.R., Plunkett G., Bloch C.A., Perna N.T., Burland V., Riley M., Collado-Vides J., Glasner J.D., Rode C.K., Mayhew G.F. (1997). The Complete Genome Sequence of Escherichia Coli K-12. Science.

[161] Van der Heijden I., Gomez-Eerland R., van den Berg J.H., Oosterhuis K., Schumacher T.N., Haanen J.B.A.G., Beijnen J.H., Nuijen B. (2013). Transposon Leads to Contamination of Clinical PDNA Vaccine. Vaccine.

